# Near‐Infrared Bodipy‐Based Molecular Rotors for β‐Amyloid Imaging In Vivo

**DOI:** 10.1002/adhm.202300733

**Published:** 2023-08-27

**Authors:** Lijun Ma, Yujie Geng, Guoyang Zhang, Ziwei Hu, Tony D. James, Xuefei Wang, Zhuo Wang

**Affiliations:** ^1^ State Key Laboratory of Chemical Resource Engineering College of Chemistry Beijing Advanced Innovation Center for Soft Matter Science and Engineering Beijing University of Chemical Technology Beijing 100029 China; ^2^ Department of Chemistry University of Bath Bath BA2 7AY UK; ^3^ School of Chemistry and Chemical Engineering Henan Normal University Xinxiang 453007 China; ^4^ School of Chemistry and Chemical Engineering University of Chinese Academy of Sciences Beijing 100049 China

**Keywords:** β‐amyloid, biological imaging, central nervous system, fluorescent probes, rodent models

## Abstract

β‐amyloid (Aβ) is one of the important biomarkers for diagnosing Alzheimer's disease (AD). Many near‐infrared probes based on the donor‐π‐acceptor structure have been developed to detect Aβ. Most reported Aβ probes are based on the *N*,*N*‐dimethylamino group as the ideal donor, which is a widely accepted binding unit. As such, the development of fluorescent probes with improved binding units to detect Aβ is urgently required. Therefore, with this research three anchoring molecular rotor electron donors consisting of cyclic amines of different ring sizes are developed, namely five‐membered ring (TPyr), six‐membered ring (TPip), and seven‐membered ring (THAI). These new anchored molecular rotors are connected to a 4,4‐difluoro‐4‐bora‐3a,4a‐diaza‐s‐indacene (BODIPY) and named TPyrBDP, TPipBDP, and THAIBDP. These probes exhibit high affinities (from 28 to 54 nm) for Aβ_1–42_ aggregates. The six‐membered ring dye TPipBDP exhibits the highest signal‐to‐noise (75.5‐fold) and higher affinity (28.30 ± 5.94 nm). TPipBDP can cross the blood‐brain barrier and exhibits higher fluorescence enhancement with APP/PS1 (AD) double transgenic (Tg) mice than with wild‐type (WT) mice.

## Graphical abstract

This research develops new BODIPY‐based probes with five, six, and seven‐membered cyclic amine structures for binding Aβ selectively. The cyclic amines can improve the electron‐donating ability, the stokes shift, and the imaging signal‐to‐noise ratio of the fluorescent probes.   The Aβ‐targeted imaging profile of the BODIPY probes with cyclic amines is evaluated in vitro and in vivo.

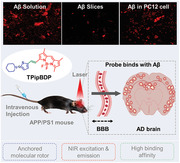

## 1 Introduction

Neurodegeneration causes the gradual loss of neuronal structure and function,^[^ ^1^ ^]^ which can lead to cognitive impairments such as depression,^[^ ^2^ ^]^ schizophrenia,^[^ ^3^ ^]^ and dementia.^[^ ^4^ ^]^ Alzheimer's disease (AD) is a neurodegenerative disease, where with an increase of age, the incidence of AD increases year on year.^[^ ^5^, ^6^ ^]^ According to the amyloid cascade hypothesis, β‐amyloid (Aβ) is believed to be responsible for AD.^[^ ^7^ ^]^ Aβ is cleaved from β‐precursor protein (APP) into fragments of 39–43 amino acids in length by a proteolytic pathway.^[^ ^8^ ^]^ Aβ includes many subtypes, the most common subtypes of Aβ in the human body are Aβ_1–40_ and Aβ_1–42_.^[^ ^9^ ^]^ Aβ tends to aggregate, from Aβ monomers, Aβ oligomers, to Aβ aggregates.^[^ ^10^ ^]^ Both Aβ_1–40_ and Aβ_1–42_ are produced by the sequential cleavage of APP by β‐secretase and γ‐secretase.^[^ ^11^ ^]^ Aβ_1–42_ has greater neurotoxicity and exhibits remarkably faster aggregation than Aβ_1–40_.^[^ ^12^ ^]^ The peptide fragment Aβ_16–21_ (KLVFFA) is highly hydrophobic and is believed to be the central hydrophobic core (CHC) of Aβ_1–42_ aggregates.^[^ ^13^, ^14^, ^15^ ^]^ Dimerization starts with the CHC unit and the process continues with attachment of another monomer to form an antiparallel β‐sheet. Theβ‐sheets then induce aggregation and deposition of Aβ_1–42_ forming senile plaques (SP).

The blood‐brain barrier (BBB) is an important barrier for maintaining the homeostasis of the brain's internal environment. It can prevent about 98% of small molecules and almost 100% of large molecules from entering, thereby protecting thebrain.^[^ ^16^ ^]^ The BBB is connected by brain capillary endothelial cells (BCECS), pericytes, astrocytes, and neuronal cells.^[^ ^17^ ^]^ Aβ_1–42_ usually accumulates in the hippocampus and cortex of brains, and the imaging and diagnosis of Aβ_1–42_ in vivo are difficult due to the existence of the BBB.^[^ ^18^, ^19^ ^]^ As such the influence of the BBB needs to be considered when designing fluorescent probes for brain imaging. Generally, probes with log *P* values between 2 and 5 and molecular weights less than 500 Da coupled with near‐infrared (NIR) emission wavelengths are essential to penetrate the BBB and facilitate brain imaging in vivo.^[^ ^20^ ^]^

Positron emission tomography (PET) and single‐photon emission computed tomography (SPECT) are two common clinical methods for the diagnosis of AD. However, these two methods require suitable radionuclide‐labeled probes as imaging agents and transfer radioactive elements into the human body, which can cause certain risks.^[^ ^21^ ^]^ Compared with these two methods, optical imaging does not require radionuclide‐labeled probes and can image the brain in vivo in real time. Especially, fluorescent probes with NIR emission (650–900 nm) exhibit deeper imaging depth and have been extensively studied over recent years.^[^ ^22^, ^23^, ^24^, ^25^ ^]^

**Scheme 1:**
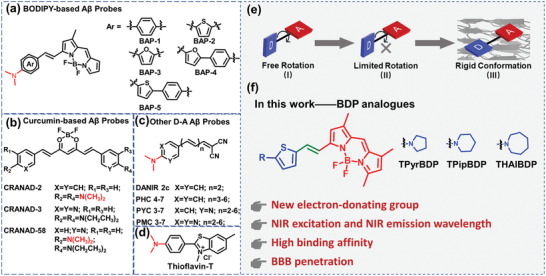
Chemical structures of reported a) BODIPY‐based Aβ probes, b) curcumin‐based Aβ probes, and c) other D–A type Aβ probes as NIR probes for the imaging of Aβ. d) The chemical structure of thioflavin‐T. e) Schematic illustration of the conformational change when the probe binds to Aβ aggregates. f) Schematic illustration of our rationally designed BDPs for Aβ plaques based on the donor‐acceptor strategy.

Recently, many fluorescent probes such as 4,4‐difluoro‐4‐bora‐3a,4a‐diaza‐s‐indacene (BODIPY) analogs,^[^ ^26^ ^]^ curcumin analogs,^[^ ^27^ ^]^ other donor‐acceptor (D–A) type probes^[^ ^28^ ^]^ have been reported for the imaging of Aβ (**Scheme** **1**). Organo difluoroboron complexes are a class of important fluorescent probes, broadly used in bioimaging fields. BODIPY‐based probes are one class of organo difluoroboron complexes with many advantages such as a high molar extinction coefficient, high fluorescence quantum yield, and good photostability. Some BODIPY‐based probes have been used for the imaging of Aβ in vivo.^[^ ^29^, ^30^ ^]^ In particular, BAP‐1 exhibits an excellent affinity for Aβ aggregates (Scheme 1a). However, the small stokes shift of BAP‐1 is not good for in vivo applications.^[^ ^31^ ^]^ Curcumin derivatives are another class of organo difluoroboron complexes used for the detection of Aβ (Scheme 1b). The curcumin derivatives have boron atoms coordinated with oxygen atoms, not nitrogen atoms.^[^ ^32^ ^]^ Based on the intramolecular charge transfer (ICT) effect, DANIR 2c and its analogs have been developed as Aβ probes (Scheme 1c).^[^ ^33^ ^]^ Thioflavin‐T (ThT) is the gold‐standard commercial Aβ probe, commonly used for staining Aβ brain slices to confirm the existence of Aβ (Scheme 1d).^[^ ^34^ ^]^ These probes are all based on a molecular rotator structure and have a rotatable *N*,*N*‐dimethylamino as an electron‐donating group. These probes are structurally composed of electron donors and acceptors, which are conjugatively linked facilitating ICT. The molecular rotor *N*,*N*‐dimethylamino has been widely used in probe design since the strong electron‐donating ability and recognition of Aβ.^[^ ^35^ ^]^ Compared to *N,N*‐dimethylamino, cyclic amines have a more rigid structure. A rigid planar structure is beneficial for improving the fluorescence intensity of the probe and response to Aβ.^[^ ^36^, ^37^, ^38^, ^39^, ^40^ ^]^ The spatial conformation and steric hindrance of the cyclic amine with Aβ cause the different fluorescence responses.^[^ ^37^ ^]^ The binding between the probe and Aβ is mainly through hydrophobic interactions. As the size of the alkyl groups increases, the hydrophobicity of the probe gradually increases (Table S1, Supporting Information). The different hydrophobicity of the probes will affect their binding to Aβ. The spatial conformation and steric hindrance of the cyclic amine lead to different fluorescence responses after binding with Aβ. We replaced *N,N*‐dimethylamino with five, six, and seven‐membered cyclic amine structures to improve the electron‐donating ability, the stokes shift, and the imaging signal‐to‐noise ratio. Herein, we investigate the effect of the size of the alkyl groups, steric hindrance, and rigid cyclic amines with Aβ. To the best of our knowledge, there has been no systematic study on the BODIPY probes with cyclic alkyl amine structures as anchored molecular rotors for the recognition of Aβ. With this research, a series of anchoring molecular rotors based on *N*,*N*‐dimethylamino namely TPyr (five‐membered ring), TPip (six‐membered ring), and THAI (seven‐membered ring) have been developed to generate Aβ probes with high affinity. These molecular rotors were linked with a thiophene group to serve as electron‐donor groups. The electron‐donor groups were then conjugated with BODIPY derivatives, namely TPyrBDP, TPipBDP, and THAIBDP, respectively. Gaussian calculations confirmed that the three new probes were ICT‐based probes. Molecular docking studies indicated that the three probes exhibited the same binding sites with Aβ_1–42_ aggregates. The ICT effect and the molecular docking results of the probes confirmed the ability for sensing Aβ. In the following experiments, we evaluated the fluorescence response and binding affinities (Table S1, Supporting Information) of the three probes and found that the six‐membered ring dye TPipBDP exhibited the highest signal‐to‐noise (75.5‐fold) and higher affinity (28.30 ± 5.94 nm). In vivo fluorescence imaging indicates that TPipBDP can cross the BBB and exhibits a higher fluorescence enhancement for APP/PS1 (AD) double transgenic (Tg) mice than that of wild‐type (WT) mice.

## 2 Results and Discussion

### 2.1 Design, Theoretical Calculations, and Synthesis of Probes

BODIPY‐based probes have many advantages such as high quantum yields, excellent stability, and good biocompatibility, and are suitable for in vivo applications. ICT is a common strategy for designing NIR‐based probes. A strong electron‐donating group and strong electron‐withdrawing group are connected through a conjugated structure to form a push‐pull structure, which can easily move the emission wavelength of the molecule to the NIR region. In addition, the electron cloud distribution of the highest occupied molecular orbital (HOMO) and the lowest unoccupied molecular orbital (LUMO) of ICT molecules results in a large stokes shift, which is beneficial for biological imaging. The boron atom in BODIPY is an electron‐deficient unit that readily accepts electrons. Meanwhile, the fluorine atom is an atom with strong electronegativity. Therefore, the backbone of BODIPY is an excellent electron‐withdrawing unit on which to construct ICT‐based probes. Therefore, to provide strong electron‐donating groups, a series of *N*,*N*‐dimethylamino derivatives (TPyr, TPip, THAI) were chosen as anchoring molecular rotors to provide electron donors, and thiophene was introduced to enhance the electron‐donating ability. Probes for the detection of Aβ usually generate fluorescence enhancement, since the probe is inserted into the hydrophobic cavity of Aβ, and rotation of the probe is inhibited, which results in enhanced fluorescence. Molecular rotor‐based probes have freely rotatable single bonds, the probes can rotate freely in solution, and part of their energy can be dissipated through this motion. When the rotation of the probe is inhibited, the probe exists in a fixed and anchored conformation, resulting in enhanced fluorescence (Scheme 1e). The introduction of a double bond can increase the length of conjugation in the system and as such promote a redshift of the probes emission wavelength. In addition, donor and acceptor groups at the ends of the double bond tend to produce a distorted molecular conformation due to the free rotation of the single bonds and steric effects of the substituents. Therefore, the existence of a double bond in a molecule provides the probe with a twisted conformation and redshifts the emission wavelength.^[^ ^41^, ^42^, ^43^ ^]^

**Figure 1:**
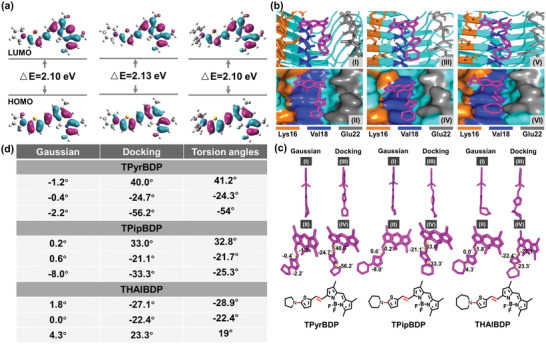
The theoretical calculation of the probes. a) The frontier molecular orbitals of the probes TPyrBDP (left), TPipBDP (middle), and THAIBDP (right). b) The docking results between BDPs and Aβ_1–42_ aggregates. I) Licorice form of TPyrBDP in the Aβ_1–42_ aggregate model. II) Molecular surface form of TPyrBDP in the Aβ_1–42_ aggregate model. III) Licorice form of TPipBDP in the Aβ_1–42_ aggregate model. IV) Molecular surface form of TPipBDP in the Aβ_1–42_ aggregate model. V) Licorice form of THAIBDP in the Aβ_1–42_ aggregate model. VI) Molecular surface form of THAIBDP in the Aβ_1–42_ aggregate model. c) Changes of dihedral angles before and after docking. I,II) The Gaussian configuration optimization and dihedral angle of TPyrBDP (left), TPipBDP (middle), and THAIBDP (right). III,IV) Corresponding molecular conformation and dihedral angle changes after molecular docking. d) Table of dihedral angles and torsion angles corresponding to c; a positive number implies twisted outward, and negative number implies twisted inward.

We replaced the *N*,*N*‐dimethylamino group with cyclic amine groups (five‐, six‐, and seven‐membered) as molecular rotors for binding Aβ. To enhance the electron‐donating ability of the electron‐donating group, thiophene was introduced as an electron‐donating group (Scheme 1f). To study the ICT effect of the three probes, Gaussian calculations were performed using density functional theory (DFT) and B3LYP/6‐311 + (d,p). As shown in **Figure** **1**a, the obtained electronic cloud distributions in the frontier orbitals revealed an electron redistribution from the thiophene unit in the HOMO to the electronic acceptor BODIPY unit in the LUMO. In addition, there were no obvious differences in the energy gap (△E). The △E of TPyrBDP, TPipBDP, and THAIBDP were 2.10, 2.13, and 2.10 eV, respectively (Figure 1a). The Gaussian theoretical calculation clearly illustrated the existence of an ICT effect in all three probes.

To predict the interaction of the probes and Aβ_1–42_ aggregates, we performed molecular docking. 5KK3 is a popular Aβ_1–42_ aggregate protein model, reported by M. T. Colvin, et al.^[^ ^44^ ^]^ The fibril core of 5KK3 consists of a Aβ_1–42_ dimer. Each molecule contains four β‐strands in an S‐shaped amyloid fold, which has two hydrophobic cores that are capped at the end of the chain by a salt bridge. 5KK3 provides sufficient β‐sheet structure to predict the interaction between the probe BDPs and Aβ_1–42_ aggregates. Therefore, the Aβ_1–42_ aggregate protein model (PDB ID: 5KK3) was chosen to evaluate the interaction between the probes and Aβ_1–42_ aggregates. The optimized structures by Gaussian were used as input files for docking. The results were processed using Autodock Tools 1.5.6, PyMOL, and Python software, which are shown in the licorice form and molecular surface form. Amino residues 17–21 and 33–42 are highly hydrophobic and constitute the hydrophobic region of Aβ_1–42_ aggregates. Therefore, these regions can provide hydrophobic pocket structures for molecular docking studies. The root‐mean‐squared deviation (RMSD) is a widely used measure of distance between two aligned objects.^[^ ^45^ ^]^ The smaller RMSD value, the better docking results. Therefore, the RMSD values of the three probes were calculated using PyMOL software. The RMSD values of TPyrBDP, TpipBDP, and THAIBDP were 0.836, 0.973, and 0.967 Å, respectively. The results indicated the docking results had good accuracy. Figures S19–S21, Supporting Information, illustrate a full view of the docking results, and Figure 1b shows a partial enlargement. In Figure 1b, TPyrBDP, TPipBDP, and THAIBDP occupied the same binding region with amino residues Lys16, Val18, and Glu22. Amongst the three amino residues, Lys16 (K16) and Val18 (V18) are components of KLVFFA. Therefore, the three probes bind with the hydrophobic core site and are inserted into the hydrophobic pocket of Aβ_1–42_ aggregates. However, the binding energy was somewhat different. The binding energy of TPyrBDP, TPipBDP, and THAIBDP were −6.46, −6.87, and −6.78 kcal mol^−1^, respectively. Among the three probes, TPipBDP exhibits the smallest value. As such, TPipBDP may exhibit a stronger binding affinity for Aβ_1–42_ aggregates than the other probes. The change in molecular dihedral angle before and after docking is shown in Figure 1c,d. The molecules after Gaussian optimization exhibit a near‐flat structure. The dihedral angles of the two adjacent faces are twisted by no more than 10 degrees. The dihedral angle increases when the molecule is inserted into the hydrophobic pocket of Aβ. As such, the changes in dihedral angle before and after docking may cause changes in fluorescence intensity. From the perspective of structural‐activity relationships (SAR), the fluorescence intensity is mainly related to the following factors. 1) The conjugated double bond (π‐bond) structure is beneficial for improving fluorescence intensity; 2) the rigid planar structure is beneficial for improving fluorescence intensity; 3) the electron donating group can increase fluorescence intensity due to p‐π conjugation. The three probes have largely conjugated structures with the same electron‐donating group. The difference among the three probes is the different cyclic amine structures. The rigid planar structure is responsible for fluorescence intensity. As shown in Figure 1c(I,III),d, the dihedral angle of TPyrBDP is close to 0° (a rigid planar structure). While after docking, the dihedral twist is ≈24.3–54°, which makes TPyrBDP to be noncoplanar. The Gaussian‐optimized dihedral angle of TPipBDP and THAIBDP is also close to 0°. After docking, the two probes' twist angle is similar. The main difference between TPipBDP and THAIBDP is the spatial distribution of cyclic amines. As shown in Figure 1c(I,III), six‐membered cyclic amines of TPipBDP exhibit good planarity with BODIPY and thiophene, while the alkyl groups of seven‐membered cyclic amines  find difficulty maintaining coplanarity with BODIPY and thiophene. So TPipBDP has better planarity. The fluorescence enhancement of TPipBDP with Aβ is higher than the other two probes. The theoretical calculation confirmed that the three probes have the possibility to interact with Aβ_1–42_ aggregates, therefore we synthesized these probes and evaluated their ability to recognize Aβ in vivo.

TPyrBDP, TPipBDP, and THAIBDP were synthesized starting with the reaction of BDP and the corresponding aldehyde, as shown in Scheme S1, Supporting Information. Briefly, BDP and compound 1 (for TPyrBDP), 2 (for TPipBDP), or 3 (for THAIBDP) were used as starting materials. The target compounds BDPs were prepared using the Knoevenagel reaction, with a catalytic quantity of piperidine and glacial acetic acid as catalysts. Detailed synthetic methods are available in the supporting information. All the compounds were characterized by ^1^H NMR, ^13^C NMR, and high‐resolution mass spectrometry (HRMS).

### 2.2 Spectral Properties of Probes and Their Response to Aβ_1–42_ Aggregates

**Figure 2:**
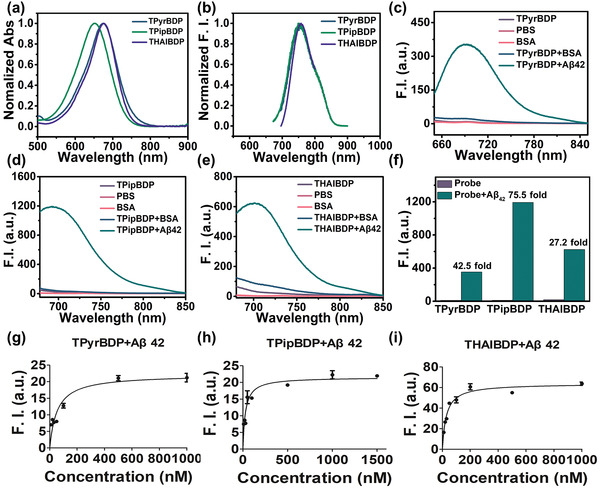
The optical properties of TPyrBDP, TPipBDP, and THAIBDP, and their fluorescence response to Aβ_1–42_ aggregates. a) The normalized absorbance of TPyrBDP, TPipBDP, and THAIBDP in DMSO solution. b) The normalized emission spectra of TPyrBDP, TPipBDP, and THAIBDP in DMSO solution. The fluorescence response of c) TPyrBDP, d) TPipBDP, e) THAIBDP to Aβ aggregates (33 µg mL^−1^, final concentration) or BSA (10 µg mL^−1^, final concentration). F.I. refers to fluorescence intensity. f) Fluorescence enhancement of BDPs. Saturated binding assay curve of g) TPyrBDP, h) TPipBDP, i) THAIBDP to Aβ_1–42_ aggregates (33 µg mL^−1^, final concentration) in different concentrations. All samples were evaluated in triplicate.

The optical properties of TPyrBDP, TPipBDP, and THAIBDP were evaluated in different solvents. We chose five solvents with different polarities to study the solvent effect of the three probes. The max absorbance wavelengths (*λ*_ex_, in DMSO) of TPyrBDP, TPipBDP, and THAIBDP were 675, 653, and 676 nm, respectively (**Figure** **2**a). The max emission wavelengths (*λ*_em_, in DMSO) of TPyrBDP, TPipBDP, and THAIBDP were 752, 751, and 758 nm, respectively (Figure 2b). As shown in Table S1 and Figure S23, Supporting Information, with an increase of solvent polarity, the emission wavelength of the three probes moved to longer wavelengths. The *λ*_ex_ of the three probes was longer than 650 nm, and the *λ*_em_ was also longer than 750 nm. In addition, TPyrBDP, TPipBDP, and THAIBDP exhibited larger stokes shifts in MeCN (80, 96, and 81 nm, respectively) than BAP‐1 (34 nm in MeCN), which is beneficial for in vivo applications. The fluorescence intensity of the three probes was enhanced with an increase of viscosity, which was associated with the restricted rotation of the chemical bonds in viscous solvents. As shown in Figure S24, Supporting Information, the F.I. was not changed in the range of 1 to 6.65 cP (1, 2‐propanediol/water, v/v), however, the F.I. was enhanced in the range of 10.04 to 33.38 cP (1, 2‐propanediol/water, v/v).

Thioflavin‐T (ThT) is the gold standard probe, commonly used as an in vitro fluorescence stain to determine the existence of Aβ aggregates. The ThT probe has a typical D–A structure, and its HOMO and LUMO electron clouds are redistributed from the donor (HOMO) to the acceptor (LUMO), which is typical for ICT probes.^[^ ^46^ ^]^ In addition, the single bond between the benzene ring in the ThT probe and the benzothiazole salt, and the *N*,*N*‐dimethylamino group attached to the benzene ring can rotate freely, resulting in ThT becoming a molecular rotor. After ThT binds to Aβ, the rotation of the molecular rotor is restricted, and most of the electrons in the excited state return to the ground state by radiative transition, resulting in a substantial increase in fluorescence. Compared with ThT, TPyrBDP, TPipBDP, and THAIBDP also exhibit molecular rotator properties, and their fluorescence intensity can change after binding to Aβ. The fluorescence response of TPyrBDP, TPipBDP, and THAIBDP with Aβ_1–42_ aggregates and BSA was evaluated in PBS (10% ethanol) solution. The formation of Aβ_1–42_ aggregates was characterized by TEM. The morphology of Aβ_1–42_ aggregates was filamentous (Figure S25b, Supporting Information), while the morphology of Aβ_1–42_ oligomers was a point‐like structure with a minimum diameter 44 nm (Figure S25a, Supporting Information). The images confirmed that Aβ_1–42_ aggregates were prepared successfully. As shown in Figure 2c‐f and Table S1, Supporting Information, a 42.5‐fold enhancement of fluorescence was observed when TPyrBDP interacted with the Aβ_1–42_ aggregates. TPipBDP and THAIBDP exhibited 75.5‐fold, and 27.2‐fold enhancement (with Aβ_1–42_ aggregates), respectively. The results confirmed the calculation results in Figure 1. The three BDPs exhibited high signal‐to‐noise (S/N) avoiding background interference. The *λ*_em_ exhibited wavelength shifts after interacting with Aβ_1–42_ aggregates. For TPyrBDP, the *λ*_em_ exhibited a redshift wavelength (≈13 nm). For TPipBDP and THAIBDP, their *λ*_em_ exhibited a blueshift wavelength (TPipBDP: ≈27 nm; THAIBDP: ≈22 nm). In Figure 1b, the three probes are inserted into the hydrophobic pocket of Aβ_1–42_ aggregates when they interact with Aβ_1–42_ aggregates, resulting in changes of *λ*_em_. The fluorescence response of BSA with probes may cause non‐specific fluorescence enhancement, thus generating false signals. Therefore, we evaluated the interaction between the three probes and BSA. Minimal enhancement was observed for the “probes + BSA” solution, indicating low non‐specific binding. The selectivity of TPyrBDP, TPipBDP, and THAIBDP to different ions and amino acids was also evaluated. As shown in Figure S26, Supporting Information, all three probes exhibited high selectivity for Aβ_1–42_ aggregates. The ions and amino acids had no influence on the fluorescence enhancement of the probes. To quantify the binding affinity (*K*_d_) of the probes to Aβ_1–42_ aggregates, fluorescence‐based saturation binding assays were conducted using Aβ_1–42_ aggregates. The lower the *K*_d_ values, the better the affinities. As shown in Figure 2g‐i and Table S1, Supporting Information, the *K*_d_ value of TPyrBDP was 54.16 ± 5.82 nm. While, TPipBDP and THAIBDP exhibited similar *K*_d_ values, which were 28.30 ± 5.94 and 28.16 ± 3.07 nm, respectively. As such TPipBDP and THAIBDP exhibited higher affinity than TPyrBDP. While the *K*_d_ value of TPipBDP and THAIBDP were smaller than those reported for CRANAD‐2 (*K*_d_ = 38.69 nm) and BAP‐1 (*K*_d_ = 44.1 nm), and like BAP‐4 (*K*_d_ = 27 nm) and QAD‐1 (*K*_d_ = 27 nm) (Table S2, Supporting Information).

**Figure 3:**
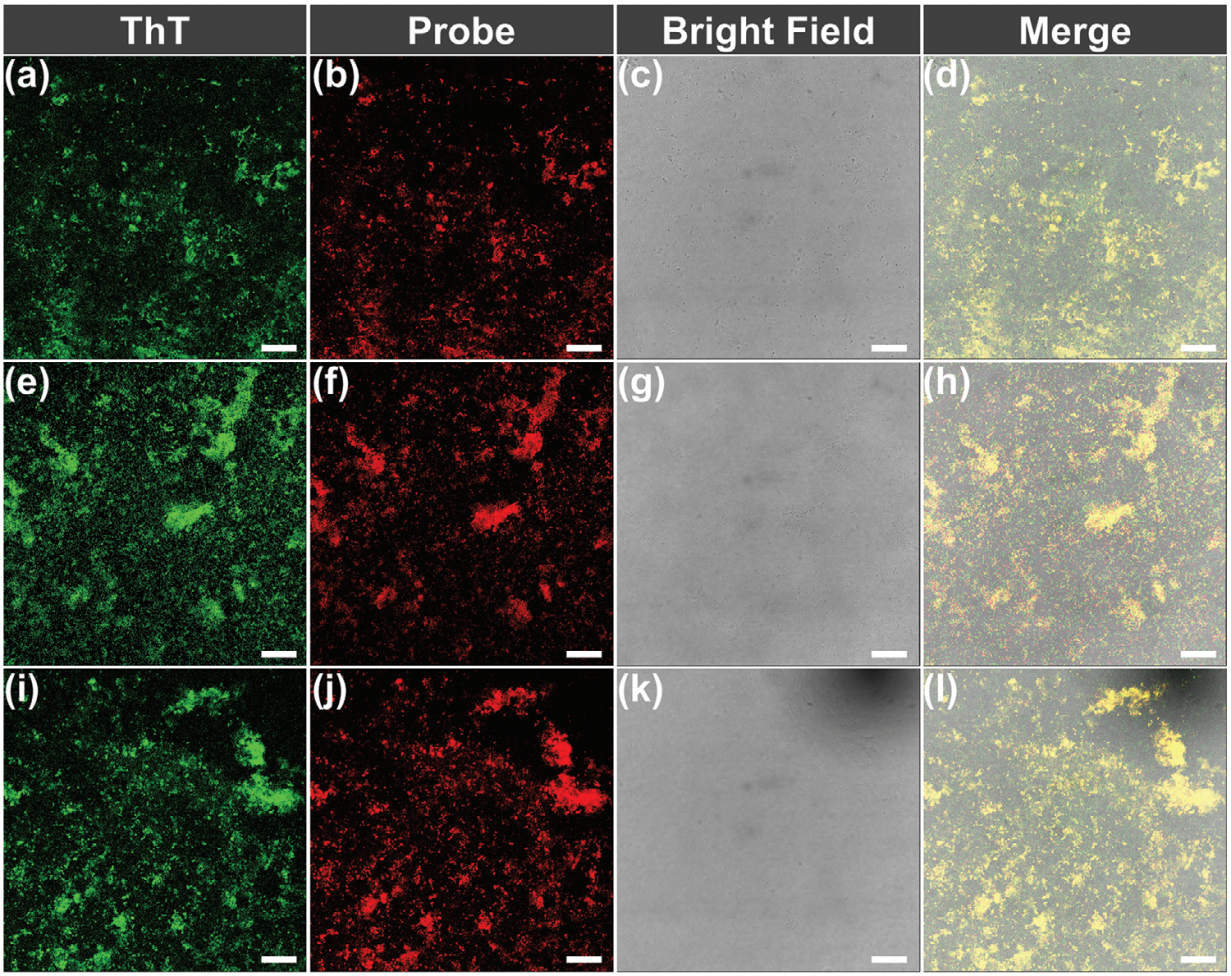
Aβ_1–42_ aggregates solution staining results using a,e,i) ThT (10 µm), b) TPyrBDP (5 µm), f) TPipBDP (5 µm), and j) THAIBDP (5 µm). c,g,k) Bright field figures. d,h,l) Merged figures. The scale bar was 5 µm.

To further assess the fluorescence changes of the probes in solution, we evaluated the staining of the probes for Aβ_1–42_ aggregates using a confocal laser scanning microscope (CLSM) in solution. Aβ_1–42_ aggregates presented plaque‐like structures under CLSM. As shown in **Figure** **3**, TPyrBDP, TPipBDP, and THAIBDP stained Aβ_1–42_ aggregates successfully and merged well with that of ThT. The results inspired us to further investigate the staining effect of the probes with Aβ brain sections.

### 2.3 In Vitro Brain Slice Staining

**Figure 4:**
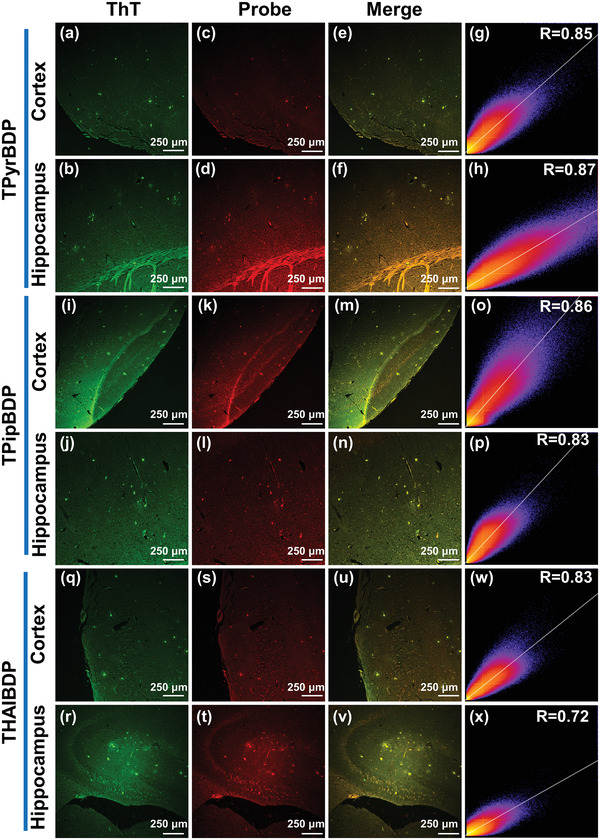
In vitro slices (13‐month old, female) staining results (10× magnification) of ThT and probes a–h) TPyrBDP, i–p) TPipBDP, and q–x) THAIBDP. a,i,q) The stain results of commercial dye ThT for the cortex region of Aβ slices. *λ*_ex_ = 488 nm, *λ*_em_ = 500–600 nm. b,j,r) The stain results of commercial probe ThT for the hippocampus region of Aβ slices. *λ*_ex_ = 488 nm, *λ*_em_ = 500–600 nm. The stain results of probes c) TPyrBDP, k) TPipBDP, and s) THAIBDP for staining the cortex region. *λ*_ex_ = 488 (60%) and 633 nm (30%), *λ*_em_ = 650–750 nm. The stain results of probes d) TPyrBDP, l) TPipBDP, and t) THAIBDP for staining the hippocampus region. *λ*_ex_ = 488 (60%) and 633 nm (30%), *λ*_em_ = 650–750 nm. Image (e,f,m,n,u,v) is the corresponding merge results. Image (g,h,o,p,w,x) is the Pearson correlation coefficient (R‐value) processed by Image*J* software.

To confirm whether the probes could bind Aβ deposits in brain slices, the fluorescence staining experiment were performed using slices of Tg mice (13‐month‐old, female). The staining results were imaged using CLSM. To ensure the accuracy of the staining results, commercial fluorescent dye ThT was used to co‐stain the same sections. The fluorescence signals of the slices were acquired at the same position using different channels of the CLSM. As shown in **Figure** **4** and Figure S27–S29, Supporting Information, Aβ plaques were highlighted by the probes with a high signal‐to‐background ratio. In contrast, there was no fluorescence signal for wild‐type slices (Figures S30–S32, Supporting Information). Since Aβ plaques are abundantly produced in the hippocampal and cortical regions of the brain, we imaged the corresponding regions of the slices, respectively. The staining results with ThT (Figure 4a,b,i,j,q,r) are shown in green, and the staining results of the probes TPyrBDP (Figure 4c,d), TPipBDP (Figure 4k,l), and THAIBDP (Figure 4s,t) are shown in red. All three probes could stain Aβ plaques both in the hippocampal and cortical regions successfully, and the corresponding merged image further confirmed the staining results. To quantify the colocalization effect, the Pearson correlation coefficients (R‐value) were calculated using Image*J* software. The closer the R‐value is to 1, the better the colocalization of the probe and ThT. As shown in Figure 4g,h, the R‐value of the TPyrBDP stained cortex region and hippocampus region were calculated as 0.85 and 0.87, respectively. The results confirmed that TPyrBDP exhibits good affinity for Aβ plaques. TPipBDP (Figure 4o,p) and THAIBDP (Figure 4w,x) also exhibited good staining results. While the R‐value of the hippocampus region stained using THAIBDP was lower than TPyrBDP and TPipBDP, the R‐value (R = 0.72) also illustrated a good affinity for Aβ plaques.

### 2.4 Biocompatibility Assessment and Cell Imaging

**Figure 5:**
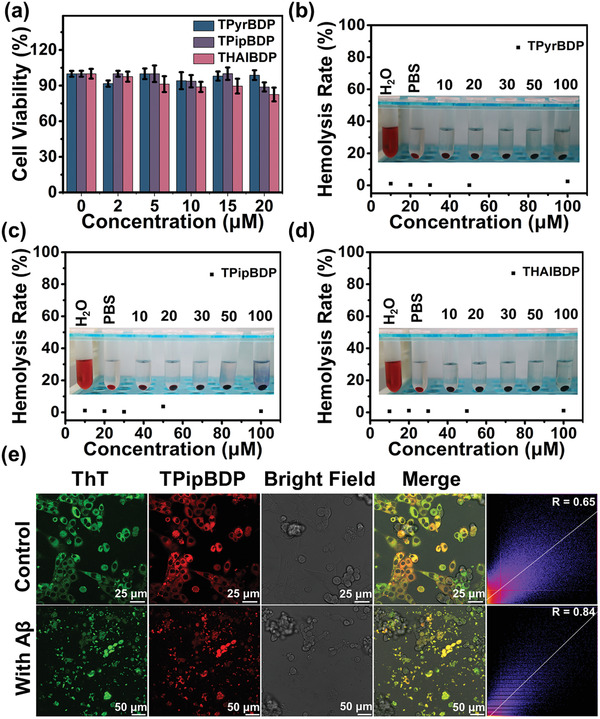
The biocompatibility and cell imaging of TPyrBDP, TPipBDP, and THAIBDP. The cytotoxicity of a) TPyrBDP, TPipBDP, and THAIBDP. The hemolysis rate of b) TPyrBDP, c) TpipBDP, d) THAIBDP. e) Imaging of Aβ_1–42_ aggregates (10 µm) in PC12 cells using TPipBDP. The last column is the Pearson correlation coefficient (R‐value) processed by Image*J* software.

A cytotoxicity assay is one of the methods to evaluate the biocompatibility of probe molecules. Probes are generally considered to have low cytotoxicity when the cell viability is above 80%. Therefore, the cytotoxicity of TPyrBDP, TPipBDP, and THAIBDP was evaluated using PC12 cells using a standard MTT assay method. As shown in **Figure** **5**a, after co‐incubating the probes TPyrBDP, TPipBDP, and THAIBDP with PC12 cells for 24 hours, the cell viability was still above 80%. The hemolysis rate of probes is one of the common methods to evaluate the effect of probes on the blood of live animals. Therefore, the hemolysis rate of probes TPyrBDP, TPipBDP, and THAIBDP with blood samples was also evaluated. As shown in Figure 5b–d, even if the concentration of the probes were as high as 100 µm, the hemolysis rate of the probes were all less than 5%, indicating that the probes exhibit a low hemolysis rate. The above results confirmed that the probes exhibit low cytotoxicity at 20 µm, and the probe exhibits a low hemolysis rate up to 100 µm. Therefore, the probes were suitable for subsequent cell experiments and animal experiments.

Since TPipBDP exhibits the best response to Aβ_1–42_ aggregates and a very low *K*_d_ value, we chose TPipBDP for the following cell and animal experiments. PC12 cells were used to study the affinity between TPipBDP and Aβ_1–42_ aggregates. Aβ_1–42_ aggregates have great neurotoxicity to interfere with the normal function of the brain. As such, Aβ_1–42_ aggregates preferentially accumulate in the cell membrane of PC12 cells, and these aggregates exhibit cytotoxicity.^[^ ^47^ ^]^ To study the imaging of TPipBDP with Aβ_1–42_ aggregates, the imaging effect of TPipBDP with Aβ_1–42_ aggregates was studied in PC12 cells. As shown in Figure 5e, TPipBDP and the commercial dye ThT exhibited the same staining site for PC12 cells in the control group. After adding Aβ_1–42_ aggregates to PC12 cells, some smaller‐than‐cell green fluorescence (ThT) and red fluorescence (TPipBDP) spots were observed. These smaller‐than‐cell bright fluorescence spots were Aβ_1–42_ aggregates. The Aβ_1–42_ aggregates can be clearly observed in the bright field image. The cell viability of the Aβ_1–42_ aggregate‐incubated PC12 cells was lower than the non‐Aβ_1–42_ aggregate‐incubated PC12 cells, which was consistent with the cytotoxicity in Figure S33, Supporting Information. Both ThT‐incubated and TPipBDP‐incubated PC12 cells exhibited the same staining site, and the R‐value of the control group and “probe + Aβ” group was 0.65 and 0.84, respectively. The results indicated that TPipBDP could image Aβ_1–42_ aggregates in PC12 cells.

### 2.5 In Vivo Fluorescence Imaging

As determined above TPipBDP exhibited the highest affinity and F.I. enhancement. Therefore, the in vivo NIR fluorescence imaging was performed to evaluate the ability of TPipBDP to penetrate the BBB and image Aβ at the same time. Before performing the experiments, we calculated the clog *P* of TPipBDP using the online ALOGPS2.1 system. The clog *P* of TPipBDP was calculated as 3.60 (Table S1, Supporting Information). Generally, probes with log P values between 2 and 5 and molecular weights less than 500 Da coupled with NIR emission wavelength are essential to penetrate the BBB and facilitate brain imaging in vivo. TPipBDP satisfies these three conditions, confirming that TPipBDP could penetrate the BBB and image Aβ in vivo. To qualitatively evaluate whether TPipBDP could penetrate the BBB, the probe TPipBDP was intravenously injected into C57BL/6J (WT mice), and the brain was dissected. The fluorescence signal of the brain was obtained using an in vivo image system (IVIS). As shown in Figure S34, Supporting Information, a fluorescence signal was observed. The results illustrated that TPipBDP could penetrate the BBB. To quantify the BBB penetration rate, HPLC was used to determine the concentration of TPipBDP in the brain. As shown in Figure S35 and Table S3, Supporting Information, the BBB penetration rate was calculated as 10.38 ± 4.11% ID/g. These results confirmed that TPipBDP could efficiently cross the BBB.

Tg mice can express a mutant human presenilin (DeltaE9) and human‐mouse amyloid preprotein (APPswe) fusion, and the expression of both genes is initiated by the mouse prion protein promoter. The DeltaE9 mutation in the human presenilin gene is caused by the deletion of the ninth exon of the gene. This mutation can cause early‐onset AD. APP/PS1 (AD) double‐transgenic mice aged 10 months will form Aβ deposits in the brain and are an ideal animal model for evaluating the deposition of Aβ in the brain.

**Figure 6:**
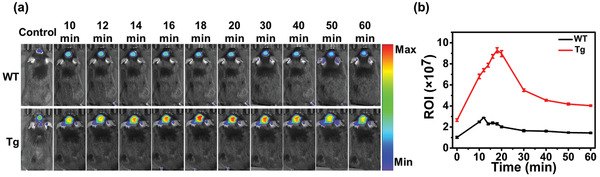
a) In vivo fluorescence imaging in the brain region of the wild‐type mice (WT, C57BL/6J, 10 months old, female) and transgenic mice (Tg, APP/PS1, 10 months old, female). TPipBDP (0.15 mg kg^−1^ in 50% PBS and 50% 1,2‐propanediol) was injected intravenously into the mice, and the fluorescence signal was obtained by an IVIS. b) Clearance curve of TPipBDP in the Tg and WT mice; the results were determined from the values of the ROI value. The unit of ROI is [(p s^−1^ cm^−2^ sr^−1^)/(µW cm^−2^)].

As shown in **Figure** **6**a, both the WT mice and Tg mice exhibited stronger fluorescence signals than the control mice, indicating that TPipBDP had crossed the BBB successfully. The fluorescence signals of WT mice were not obviously changed as time elapsed. In contrast, the fluorescence signals of Tg mice exhibited obvious intensity changes. The fluorescence signal of Tg mice was consistently higher than that of WT mice. The Tg mice exhibited the highest fluorescence signal at 18 minutes after intravenous injection of TPipBDP. To semi‐quantify the fluorescence intensities, the region of interest (ROI) around the brain region was drawn to give relative fluorescence intensities. In Figure 6b, the metabolism curve was plotted by the ROI versus time after intravenous injection. The highest fluorescence signal of WT mice was at 12 minutes and then decreased gradually. In contrast, the highest fluorescence signal of Tg mice was at 18 minutes and then decreased gradually. The maximum fluorescence intensity of Tg mice was about three times that of WT mice, indicating that TPipBDP could bind strongly with Aβ in the brain of Tg mice. The clearance of TPipBDP in Tg mice was slower than that of WT mice. In summary, the animal experiments confirmed that TPipBDP could cross the BBB and image the Aβ in vivo.

## 3 Conclusion

We have developed three probes for imaging Aβ_1–42_ aggregates. BDPs contain different electron‐donating groups when compared to traditional *N*,*N*‐dimethylamino and exhibit enhanced redshift absorbance and emission wavelengths than BAP‐1. Gaussian calculations confirmed that the BDPs were ICT‐based probes. The molecular docking studies confirmed that the BDPs interacted with two hydrophobic amino acid residues, Lys16 (K16) and Val18 (V18) of Aβ_1–42_ aggregates and inserted into the hydrophobic pockets of Aβ_1–42_ aggregates, resulting in enhanced fluorescence. We evaluated the fluorescence response between BDPs and Aβ_1–42_ aggregates in solution, and the results confirmed the docking results. After interacting with Aβ_1–42_ aggregates, the fluorescence intensities of TPyrBDP, TPipBDP, and THAIBDP were enhanced by 42.5, 75.5, and 27.2 times, respectively. The binding affinities of TPyrBDP, TPipBDP, and THAIBDP for Aβ_1–42_ aggregates were 54.16 ± 5.82, 28.30 ± 5.94, and 28.16 ± 3.07 nm, respectively. The BDPs exhibited good staining ability for Aβ plaques in brain slices and had a good co‐localization with that of ThT. TPipBDP could image exogenous Aβ in PC12 cells. In addition, TPipBDP can penetrate the BBB and image Aβ plaques in vivo. The BDP probe with a six‐membered ring structure exhibited high S/N ratio fluorescence imaging for Aβ and exhibited a high affinity for Aβ. This research provides a new design strategy for high affinity Aβ probes which should enable a better understanding of the AD pathological processes.

## Conflict of Interest

The authors declare no conflict of interest.

## Supporting information

Supporting Information

## Data Availability

The data that support the findings of this study are available in the supplementary material of this article.
