# Supplementary material for: Near‐Infrared Bodipy‐Based Molecular Rotors for β‐Amyloid Imaging In Vivo
Source: Adv Healthc Mater. 2023 Aug 27;12(25):2300733. doi: 10.1002/adhm.202300733 (PMC11468675; doi:10.1002/adhm.202300733)
Supplement: Supplementary file 1 — Supporting Information [file ADHM-12-2300733-s001.pdf]

ADVANCED HEALTHCARE MATERIALS

Supporting Information

for *Adv. Healthcare Mater.*, DOI 10.1002/adhm.202300733

Near-Infrared Bodipy-Based Molecular Rotors for *β*-Amyloid Imaging In Vivo

Lijun Ma, Yujie Geng, Guoyang Zhang, Ziwei Hu, Tony D. James, Xuefei Wang* and Zhuo Wang**

Supporting Information

Near-Infrared BODIPY based Molecular Rotors for β-Amyloid Imaging *In Vivo*

*Lijun Ma,[a] Yujie Geng,[a] Guoyang Zhang,[a] Ziwei Hu,[a] Tony D. James,[*c,d] Xuefei Wang,[*b] Zhuo Wang[*a,c]*

a. State Key Laboratory of Chemical Resource Engineering, College of Chemistry, Beijing Advanced Innovation Center for Soft Matter Science and Engineering, Beijing University of Chemical Technology, Beijing, 100029, China. E-mail: wangzhuo77@mail.buct.edu.cn.

b. School of Chemical Sciences, University of Chinese Academy of Sciences, Beijing, 100049, P. R. China. Email: wangxf@ucas.ac.cn.

c. Department of Chemistry, University of Bath, BA2 7AY, UK.

d. School of Chemistry and Chemical Engineering, Henan Normal University, Xinxiang 453007, China. Email: T.D.James@bath.ac.uk

Table of Contents

General Information…………………………………………………………….....S4

Reagents, Materials, and Animals……………………………………………………S4

Preparation of probes…………………………………………………………………..S5

Preparation of Aβ_{42} aggregates…………………………………………………S7

Fluorescence Enhancement Assay…………………………………………………S8

Saturated Binding Assay……………………………………………………………...S8

Selectivity Assay………………………………………………………………………..S8

Docking Studies………………………………………………………………………...S9

In Vitro Aβ_{42} staining in solution…………………………………………………S9

In Vitro Brain Slice Fluorescence Staining…………………………………………S9

Hemolysis Rate (HR) Assay…………………………………………………………S10

Cytotoxicity Assay……………………………………………………………………...S10

Cell imaging…………………………………………………………………………….S11

Animal Experiments…………………………………………………………………...S11

Characterization of Probes…………………………………………………………S12

Figure S18. The molecular configuration optimized using Gaussian…………….S22

Figure S19. The overview of docking results of TPyrBDP in 5KK3 protein model..S23

Figure S20. The overview of docking results of TPyrBDP in 5KK3 protein model..S23

Figure S21. The overview of docking results of THAIBDP in 5KK3 protein model..S23

Figure S22. The absorption spectra of probes in different solvents………………..S24

Figure S23. The emission spectra of probes in different solvents……………………S24

Table S1. The spectral data of probes…………………………………………………S24

Table S2 Comparation of our probes with reported probes…………………………S25

Figure S24. The viscosity response characteristics of BDPs…………………….….S27

Table S3. The relationship between volume percentage and viscosity………………S27

Figure S25. The TEM images of Aβ……………………………………………………..S28

Figure S26. The selectivity of BDPs to different ions and Aβ_{42} aggregates………..S28

Figure S27. *In vitro* fluorescence staining results (TPyrBDP) for brain slices from Tg

mice……………………………………………………………………………S29

Figure S28. *In vitro* fluorescence staining results (TPipBDP) for brain slices from Tg mice……………………………………………………………………………S29

Figure S29. *In vitro* fluorescence staining results (THAIBDP) for brain slices from Tg mice……………………………………………………………………………S30

Figure S30. *In vitro* fluorescence staining results (TPyrBDP) for brain slices from WT mice……………………………………………………………………………S30

Figure S31. *In vitro* fluorescence staining results (TPipBDP) for brain slices from WT mice……………………………………………………………………………S31

Figure S32. *In vitro* fluorescence staining results (THAIBDP) for brain slices from WT mice……………………………………………………………………………S31

Figure S33. Cytotoxicity of $A\beta_{1\text{-}42}$ aggregates………………………………………S32

Figure S34.The biodistribution of TPipBDP after intravenous injection…………...S32

Figure S35. The BBB penetration rate of TPipBDP………………………………S33

Table S4. Uptake of TPipBDP in the brains of KM mice……………………………S33

References…………………………………………………………………………S34

General Information

Reagents, Materials, and animals. All the reagents were commercial products and used without further purification unless otherwise stated. 3,5-Dimethylpyrrole-2-carboxaldehyde (CAS no. 62199-58-8, A68010), Phosphorus oxychloride ($POCl_3$, CAS no. 10025-87-3, A67421), Boron trifluoride diethyl etherate (BF_3OEt_2, CAS no. 109-63-7, A20451), 5-Bromothiophene-2-carbaldehyde (CAS no. 4701-17-1, A20587), Acetic Acid (HAc, CAS no. 64-19-7, A27238), 4-Acetamidobenzaldehyde (CAS no. 122-85-0, A15043) were purchased from Beijing Innochem Technology Co., Ltd. Triethylamine (Et_3N, CAS no. 121-44-8, 17471H), Pyrrolidine (CAS no. 123-75-1, 18283A) were purchased from Adamas. Piperidine (CAS no. 110-89-4) were purchased from Sinopharm Chemical Reagent Co., Ltd (China). Hexamethyleneimine (CAS no. 111-49-9, B24007) was purchased from Alfa. (4-formyl-phenyl)-carbamicacidtert-butylester (CAS no. 144072-30-0, 1112669) was purchased from Shanghai Haohong Biomedical Technology Co., Ltd (China). β-amyloid (1−42) human ($A\beta_{1-42}$) were purchased from Shanghai Macklin Biochemical Technology Co., Ltd. Human recombinant alpha-synuclein pre-formed fibrils (Type 1, Cat # SPR-322B) and Tau441 (2N4R) P301S mutant pre-formed fibrils (Cat # SPR-329B) were purchased form StressMarq Biosciences, Canada. High glucose Dulbecco's modified Eagle's medium (DMEM), heat-inactivated fetal bovine serum (FBS), penicillin and streptomycin, and PBS (pH = 7.2−7.4, 0.01 M) were purchased from Hyclone. The blood sample was provided by Beijing China-Japan Friendship Hospital. The 3-(4,5-dimethylthiazol-2-yl)-2,5-diphenyltetrazolium bromide (MTT) kit was purchased from KeyGEN BioTECH (Jiangsu, China).

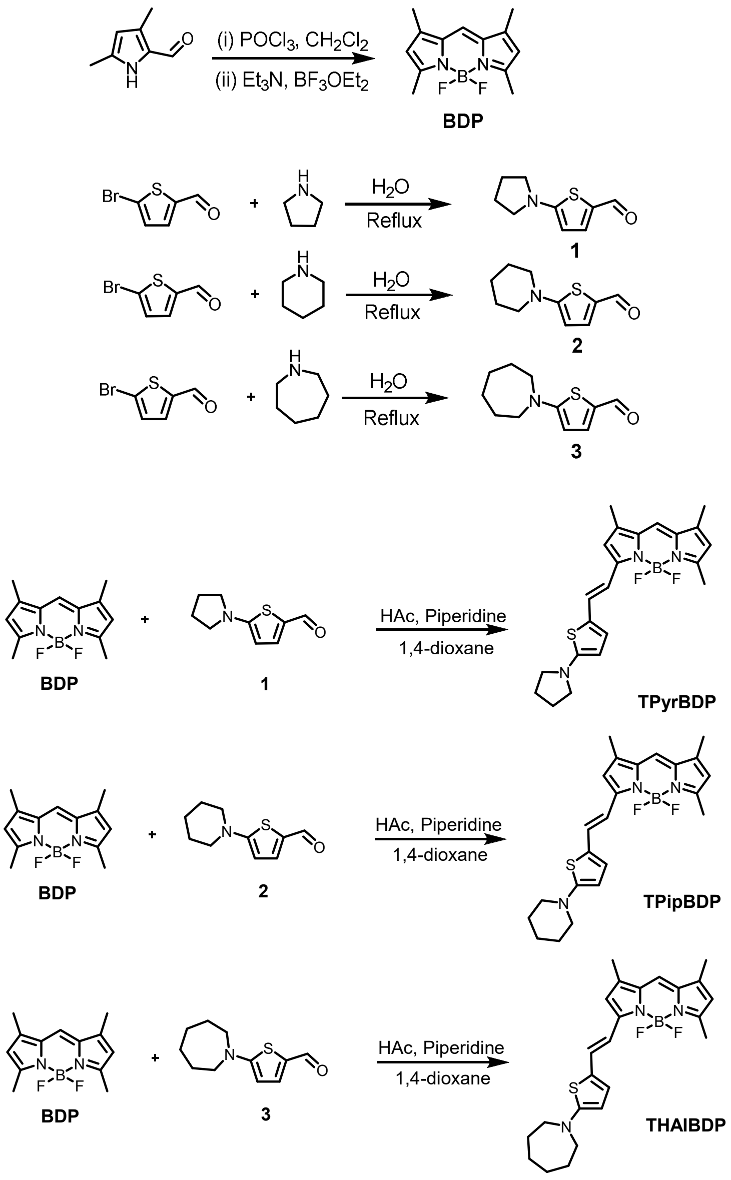

Scheme S1. The synthetic route of BODIPY derivatives.

Preparation of BDP. BDP was synthesized according to previous literature methods [1]. ^{1}H NMR (400 MHz, $CDCl_3$) δ 6.96 (s, 1H), δ 5.97 (s, 2H), δ 2.46 (s, 6H), δ 2.17 (s, 6H). EI-MS *m/z*: cacld for $C_{13}H_{15}N_2BF_2$, 248.08; found, 248.

***General procedure A*.** To a solution of 5-bromothiophene-2-carboxaldehyde (5.24 mmol, 1.00 g) in boiling water was added the corresponding amino compound (5.76 mmol). The resulting mixture was refluxed for 18 h and cooled to room temperature. The mixture was extracted by DCM (3×30 mL) and the combined organic phase was dried with anhydrous Na_2SO_4. The solvent was evaporated under reduced pressure. The crude product was purified by silica gel column chromatography (PE/EA = 5/1 ~ PE/EA

= 1/1, v/v) to give pure products.

5-(pyrrolidin-1-yl)thiophene-2-carbaldehyde (1). Compound 1 was synthesized according to *General Procedure A*. Compound 1 was obtained as pink solid with a yield of 72 % (0.68 g).^{1}H NMR (400 MHz, $CDCl_3$) δ 9.48 (s, 1H), δ 7.48 (d, *J* = 4.4 Hz, 1H), δ 5.87 (d, *J* = 4.4 Hz, 1H), δ 3.43 – 3.36 (m, 4H), δ 2.14 – 2.08 (m, 4H). EI-MS *m/z*: cacld for $C_9H_{11}NOS$, 181.05613; found, 181.05558.

5-(piperidin-1-yl)thiophene-2-carbaldehyde (2). Compound 2 was synthesized according to *General Procedure A*. Compound 2 was obtained as pink solid with a yield of 60 % (0.61 g). ^{1}H NMR (400 MHz, $CDCl_3$) δ 9.52 (s, 1H), δ 7.50 (d, *J* = 4.5 Hz, 1H), δ 6.09 (d, *J* = 4.5 Hz, 1H), δ 3.43 – 3.29 (m, 4H), δ 1.80 – 1.61 (m, 6H). EI-MS *m/z*: cacld for $C_{10}H_{13}NOS$, 195.07178; found, 195.07098.

5-(azepan-1-yl)thiophene-2-carbaldehyde (3). Compound 3 was synthesized according to *General Procedure A*. Compound 3 was obtained as pink solid with a yield of 49 % (0.54 g). ^{1}H NMR (400 MHz, $CDCl_3$) δ 9.46 (s, 1H), δ 7.46 (d, *J* = 4.4 Hz, 1H), δ 5.93 (d, *J* = 4.5 Hz, 1H), δ 3.53 – 3.45 (m, 4H), δ 1.83 (s, 4H), δ 1.60 (dt, *J* = 5.6, 2.6 Hz, 4H). EI-MS *m/z*: cacld for $C_{11}H_{15}NOS$, 209.08743; found, 209.08661.

General Procedure B. To a single-neck bottle (100 mL) with magnet, equipped with a 5 mL Dean-Stark device, were added anhydrous1,4-dioxane (10 mL), BDP (0.4 mmol, 0.1 g), corresponding aldehyde (0.4 mmol), glacial acetic acid (HAc, 0.4 mL), and piperidine (0.4 mL). The mixture was transferred to a 130-degree oil bath and refluxed for 1-2 h. The mixture was cooled to room temperature, extracted with DCM (3×50 mL) and water. The combined organic phase was dried with anhydrous Na_2SO_4. The solvent was evaporated under reduced pressure. The crude product was repeatedly purified by silica gel column chromatography (PE/EA = 3/1, v/v) to give pure products.

(E)-5,5-difluoro-1,3,9-trimethyl-7-(2-(5-(pyrrolidin-1-yl)thiophen-2-yl)vinyl)-5H-4l4,5l4-dipyrrolo[1,2-c:2',1'-f][1,3,2]diazaborinine (TPyrBDP). Compound TPyrBDP was synthesized according to *General Procedure B*. Compound TPyrBDP was obtained as a blue-green solid with a yield of 8% (13 mg). ^{1}H NMR (400 MHz, DMSO-d_6) δ 7.67 (d, *J* = 15.4 Hz, 1H), δ 7.32 (s, 1H), δ 7.16 (d, *J* = 4.2 Hz, 1H), δ 6.87

(s, 1H), δ 6.62 (d, *J* = 15.4 Hz, 1H), δ 6.08 (s, 1H), δ 5.91 (d, *J* = 4.2 Hz, 1H), δ 3.37 (t, *J* = 4.5 Hz, 4H), δ 2.41 – 2.23 (m, 9H), δ 2.05 – 2.00 (m, 4H). ^{13}C NMR (101 MHz, $CDCl_3$) δ 190.23, δ 151.97, δ 148.70, δ 138.58, δ 135.49, δ 132.19, δ 129.55, δ 124.89, δ 123.90, δ 117.98, δ 116.91, δ 115.64, δ 113.65, δ 111.77, δ 111.20, δ 47.65, δ 29.73, δ 25.43, δ 22.71, δ 14.13. HRMS (MALDI-FTICR-MS) *m/z*: cacld for $C_{22}H_{24}BF_2N_3S$, 411.174619; found 411.175059.

(E)-5,5-difluoro-1,3,9-trimethyl-7-(2-(5-(piperidin-1-yl)thiophen-2-yl)vinyl)-5H-4l4,5l4-dipyrrolo[1,2-c:2',1'-f][1,3,2]diazaborinine (TPipBDP). Compound TPipBDP was synthesized according to *General Procedure B*. Compound TPipBDP was obtained as a blue-green or dark red (metallic luster) solid with a yield of 13% (22 mg). ^{1}H NMR (400 MHz, DMSO-d_6) δ 7.64 (d, *J* = 15.6 Hz, 1H), δ 7.39 (s, 1H), δ 7.11 (d, *J* = 4.2 Hz, 1H), δ 6.87 (d, *J* = 1.2 Hz, 1H), δ 6.68 (d, *J* = 15.5 Hz, 1H), δ 6.19 (d, *J* = 4.2 Hz, 1H), δ 6.11 (s, 1H), δ 3.31 (d, *J* = 5.6 Hz, 4H), δ 2.41 (s, 3H), δ 2.27 (d, *J* = 15.3 Hz, 6H), δ 1.62 (t, *J* = 12.4 Hz, 6H). ^{13}C NMR (101 MHz, $CDCl_3$) δ 161.48, δ 156.56, δ 152.98, δ 140.84, δ 137.72, δ 135.75, δ 132.91, δ 131.83, δ 131.55, δ 117.78, δ 116.19, δ 115.69, δ 112.50, δ 104.61, δ 77.35, δ 77.23, δ 77.03, δ 76.71, δ 51.61, δ 29.71, δ 24.97, δ 23.66, δ 14.65, δ 11.34, δ 11.26. HRMS (MALDI-FTICR-MS) *m/z*: cacld for $C_{23}H_{26}BF_2N_3S$, 425.190263; found 425.190726.

(E)-3-(2-(5-(azepan-1-yl)thiophen-2-yl)vinyl)-5,5-difluoro-1,7,9-trimethyl-5H-5l4,6l4-dipyrrolo[1,2-c:2',1'-f][1,3,2]diazaborinine (THAIBDP). Compound THAIBDP was synthesized according to *General Procedure B*. Compound THAIBDP was obtained as a golden solid. Yield: 18% (31 mg). ^{1}H NMR (400 MHz, $CDCl_3$) δ 7.32 (d, J = 15.4 Hz, 1H), δ 7.00 – 6.81 (m, 3H), δ 6.59 (s, 1H), δ 6.01 (s, 1H), δ 5.79 (d, J = 4.2 Hz, 1H), δ 3.49 (t, J = 5.9 Hz, 4H), δ 2.57 (s, 3H), δ 2.26 (d, J = 7.3 Hz, 6H), δ 1.89 – 1.81 (m, 4H), δ 1.62 (hept, J = 4.9 Hz, 4H). ^{13}C NMR (101 MHz, $CDCl_3$) δ 161.56, δ 136.48, δ 136.05, δ 133.50, δ 132.34, δ 124.95, δ 117.25, δ 115.84, δ 115.24, δ 110.61, δ 101.27, δ 77.00, δ 52.43, δ 27.54, δ 14.59, δ 11.34. HRMS (MALDI-FTICR-MS) *m/z*: cacld for $C_{24}H_{28}BF_2N_3S$, 439.206000; found 439.206393.

Preparation of Aβ$_{42}$ Aggregates

Pretreatment

To a solution of HFIP (600 μL) was added human Aβ_{42} (1 mg). After that, the solution was ultrasonically treated for 2 min to destroy the oligomer probably formed, followed by placing the mixture into an ice bath for 2 h. The solution was dispensed 20 μL per centrifuge tube, dried by nitrogen, and stored at −80 °C.

Aβ_{42} Aggregates

To a centrifuge tube (containing 33 μg of Aβ_{42}) was added 540 μL of PBS and 1% hydroxylamine solution (60 μL); the solution was shaken on a shaker (300 rpm) at 37 °C for 72-96 h to form the Aβ_{42} aggregates. The Aβ_{42} aggregates were characterized by transmission electron microscopy (TEM).

Fluorescence Enhancement Assay

To a solution of probes were added pretreated Aβ_{42} aggregates (33 μg) or bovine serum albumin (BSA). The mixture (the final concentration of probes was 1μM in 10% EtOH/PBS) was immediately transferred to a fluorescence spectrophotometer (U-3900H, HITACHI, Japan) to record their spectrum (Ex slit = 10 nm, Em slit = 20 nm). The probes alone (1μM in 10% EtOH/PBS), PBS, and BSA were also tested under the same conditions. The fluorescence enhancement was calculated using the following formula: fold = the fluorescence intensity of the probe with Aβ_{42} or BSA/the fluorescence intensity of probe alone.

Saturated Binding Assay

A solution containing a gradient concentration of probes and Aβ_{42} aggregates (3 μg) in 10% EtOH/PBS were freshly prepared. The mixture was immediately transferred to a fluorescence spectrophotometer (U-3900H, HITACHI, Japan) to record their spectrum (Ex slit = 10 nm, Em slit = 20 nm). All the samples were prepared in triplicate. The obtained data was analyzed by GraphPad Prism 5.0 software, and the Kd value was calculated using nonlinear regression. The Kd was calculated according to the formula as follows.

$$Y = \frac{B_{max} \cdot X}{k_d + X}$$

where X is the concentration of probes, Y is change in fluorescence intensity, B_{max} is

the maximum specific binding has the same units as Y, Kd is the equilibrium binding constant.

Selectivity Assay

A solution (10% EtOH/H_2O) containing probes (1 μM) + various ions (20 μM), probes (1 μM) + various amino acids (20 μM), probes (1 μM) + $A\beta_{42}$ aggregates (33 μg) were freshly prepared. The mixture was immediately transferred to a fluorescence spectrophotometer (U-3900H, HITACHI, Japan) to record their spectrum (Ex slit = 10 nm, Em slit = 20 nm). The data was processed with origin 8.0 software.

Docking Studies

Docking procedures were performed using AutoDock 4.2 software. The macromolecule receptor $A\beta_{42}$ aggregates models (PDB ID 5KK3) were downloaded from https://www.pdbus.org/. The probes were optimized with Gaussian with B3LYP 6-311G(d,p) method and served as the input ligands. The $A\beta_{42}$ aggregates model was processed by adding hydrogens, computing gasteiger, respectively, and saving it as a pdbqt file. The ligand files were processed by adding hydrogens, and computing gasteiger, respectively. Nonpolar hydrogen atoms were merged and all torsions were set to be rotatable during docking. The grid box was set as 56 Å × 126 Å × 126 Å. The spacing (angstrom) was set as 0.375. The x center, y center, and z center were 15.324, -4.615, and -28.225, respectively. The number of GA runs was set as 50. The population size, the maximum number of evaluations, and the maximum number of generations were set by default. The results were analyzed by PyMoL software.

***In Vitro* $A\beta_{42}$ staining in solution**

$A\beta_{42}$ aggregates were prepared according to the literature reported method [2]. $A\beta_{42}$ aggregate solution (20 μL) was added dropwise to a slide (CITOTEST), and then spread until dry. ThT (10 μM, 20 μL) was added dropwise and spread until it was dry. Probes were added dropwise and spread until dry. Anti-fluorescence quenching reagent (10-20 μL) was added dropwise and covered with a cover slide. The fluorescence signal was captured by CLSM. λ_{ex} (ThT) = 488 nm, λ_{em} (ThT) = 508-550 nm; λ_{ex} (probe) = 633 nm,

λ_{em} (probe) = 653-750 nm.

***In Vitro* Brain Slice Fluorescence Staining**

The paraffin-embedded blank sections (5 μm) were immersed in xylene for 5 min for deparaffinization and then washed with ethanol for 2 min and water for 5 min. The brain slices were incubated with 40 μL of 10 mg/mL ThT for 5 min and washed with 50% ethanol solution for 3 min. Next, the brain slices were incubated with 40 μL of 1 μM probes for 20 min. After absorbing the residual liquid with dust-free paper, the antifluorescence attenuating agent was added dropwise, and neutral gum was used for mounting. The slices were then placed under a laser confocal microscope (Lecia SP5) for imaging.

Hemolysis Rate (HR) Assay

Human blood (1 mL, provided by China-Japan Friendly Hospital, Beijing, China) was first diluted with 2 mL of PBS solution; the sample was centrifugated for 10 min (8000 rpm) and washed with PBS 5 times to separate red blood cells from serum. Finally, the red blood cells were dispersed in 10 mL of PBS solution, from which 0.2 mL was taken, and 0.8 mL of different reagents (PBS, DI water, and 10, 20, 30, 50, and 100 μM of probes) was added. After mixing evenly, the samples were allowed to stand still for 3 h at room temperature and centrifuged (12,000 rpm, 5 min) to determine hemolysis circumstances. The supernatant (100 μL) was transferred to 96-well plates, and its ultraviolet absorption (Ex = 490 nm) was evaluated. The hemolysis rate (HR) of the red blood cells was calculated using the following formula.

$$HR=\frac{Abs_{sample}-Abs_{PBS}}{Abs_{water}-Abs_{PBS}}$$

Cytotoxicity Assay

The cells used for the cytotoxicity test were PC12, purchased from the Chinese National Infrastructure of Cell Line Resource. The experiment was divided into two parts, cell culture, and cytotoxicity test. The PC12 cells were first cultured in cell culture fluid (high-glucose DMEM/heat-inactivated FBS/ heat-inactivated HS /penicillin and streptomycin = 100: 5: 5: 1, v/v) under 5% CO_2 at 37 °C. After a period of incubation, PC12 were transferred into a sterile 96-well plate (1×10^4 cells per well) and cultured

for 24 h under 5% CO_2 at 37 °C. After that, the cell culture fluid was removed, and gradient concentrations of probes (TPyrBDP, TPipBDP, THAIBDP), Aβ in the cell culture fluid (0, 2, 5, 10, 12, and 15 μM), or probes+ Aβ in the cell culture fluid (5 μM probe + 10 μM Aβ) were added into the 96-well plate and incubated for another 24 h. After that, the gradient concentration of probes or Aβ was removed and MTT (5× MTT was diluted into 1× MTT using dilution buffer, 50 μL per well) was added; the mixture was incubated at 37 °C for 4 h. The supernatant was removed, and formazan (150 μL per well) was added; the mixture was placed on a shaker to be mixed well. The cell viability was determined by a microplate reader at 490 nm.

Cell imaging

To a centrifuge tube (containing 1 mg of $A\beta_{42}$) was added 180 μL of DMSO and 720 μL of PBS (pH = 7.2-7.4, Hyclone) and 1% hydroxylamine solution (100 μL); the solution was shaken on a shaker (300 rpm) at 37 °C for 7 days to form the $A\beta_{42}$ aggregates. Initial peptide concentrations were determined spectrophotometrically at 280 nm, using an extinction coefficient of 1280 $M^{-1}cm^{-1}$.

PC12 cells (1×10^5 cells/mL) were inoculated in a confocal plate and incubated for 24 h. $A\beta_{42}$ aggregates (20 μM, final concentration) were added to the confocal plate and incubated for 5 minutes. ThT (100 nM) and probe (100 nM) were added and incubated for 5-10 minutes, and then image $A\beta_{42}$ aggregates with a CLSM. λ_{ex} (ThT) = 488 nm, λ_{em} (ThT) = 508-550 nm; λ_{ex} (probe) = 633 nm, λ_{em} (probe) = 653-750 nm.

Animal Experiments

$APP_{SWE}/PSEN_1dE_9$ (Tg, APP/PS1, 10-month old, female) and C57BL/6J (WT, 10-month old, female) were purchased from Beijing Huafukang Bioscience Co., LTD (China). All protocols requiring the use of animals were approved by the animal care committee of China-Japan Friendly Hospital. The approval number is zryhyy12-20-10-2. For *in vivo* fluorescence imaging, the APP/PS1 mice and wild-type mice (WT) were first head-shaved to reduce the effect of hair on fluorescence imaging. Before *in vivo* fluorescence imaging, the APP/PS1 and WT mice were first placed into an imaging box to obtain background signals. Probe (TPipBDP, 60% 1,2-Propanediol/35% PBS/5%

DMSO, 0.15 mg/kg) were injected via the mouse tail vein, and the mice were transferred into an imaging box; the fluorescence signals of the brain were recorded at different time points on an IVIS Lumina III system (PerkinElmer). The mice were anesthetized under 5% isoflurane gas and 1.0 L/min oxygen flow during the imaging process. The obtained data were analyzed with Living Image Software (Living software 4.5.5), and the region of interest (ROI) value was drawn around the brain region to obtain the fluorescence intensity of the brain region.

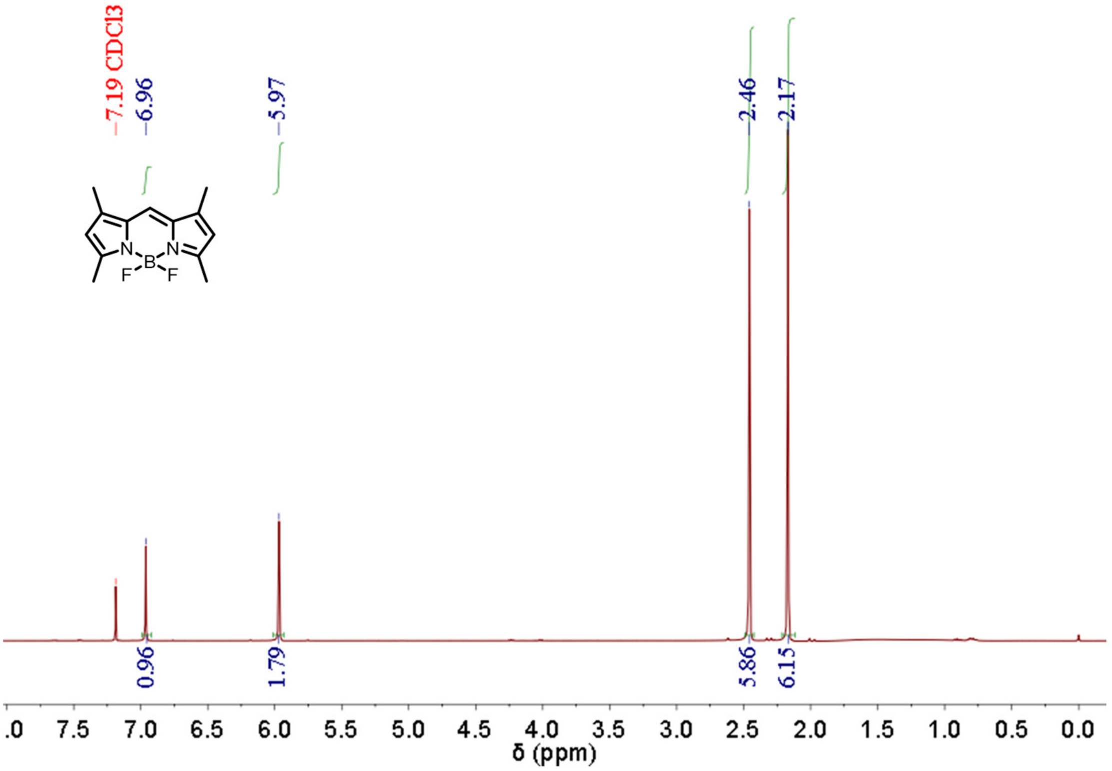

Figure S1. The ^{1}H NMR of BDP in $CDCl_3$.

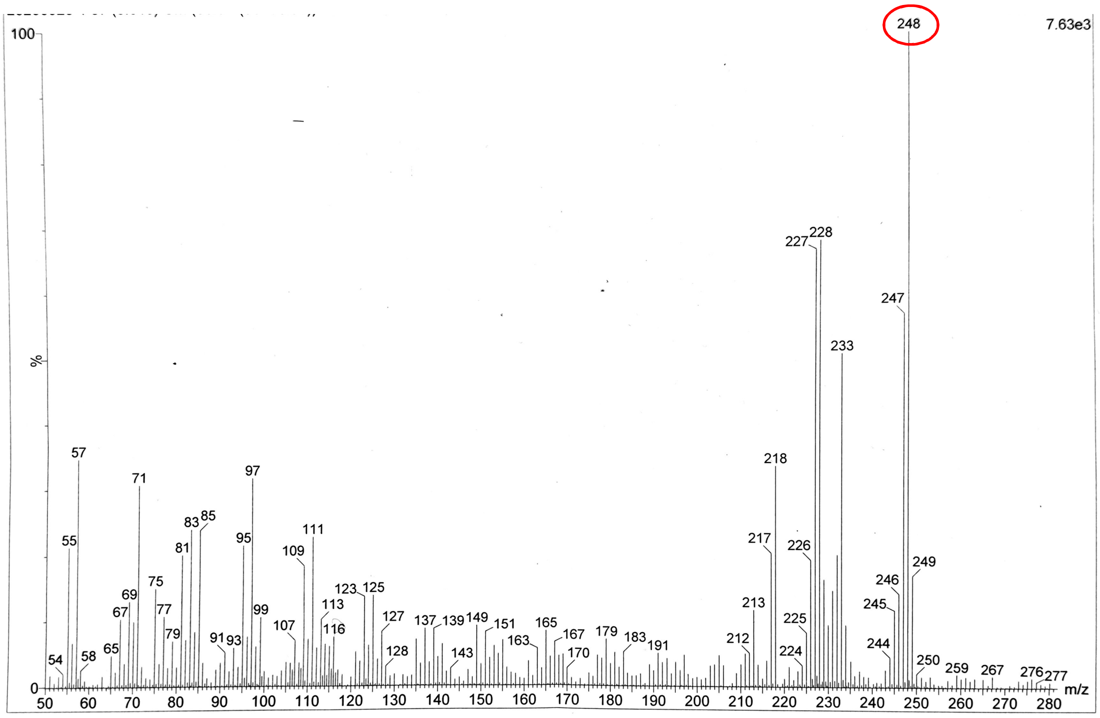

Figure S2. The mass spectrometry (EI-MS) of compound BDP.

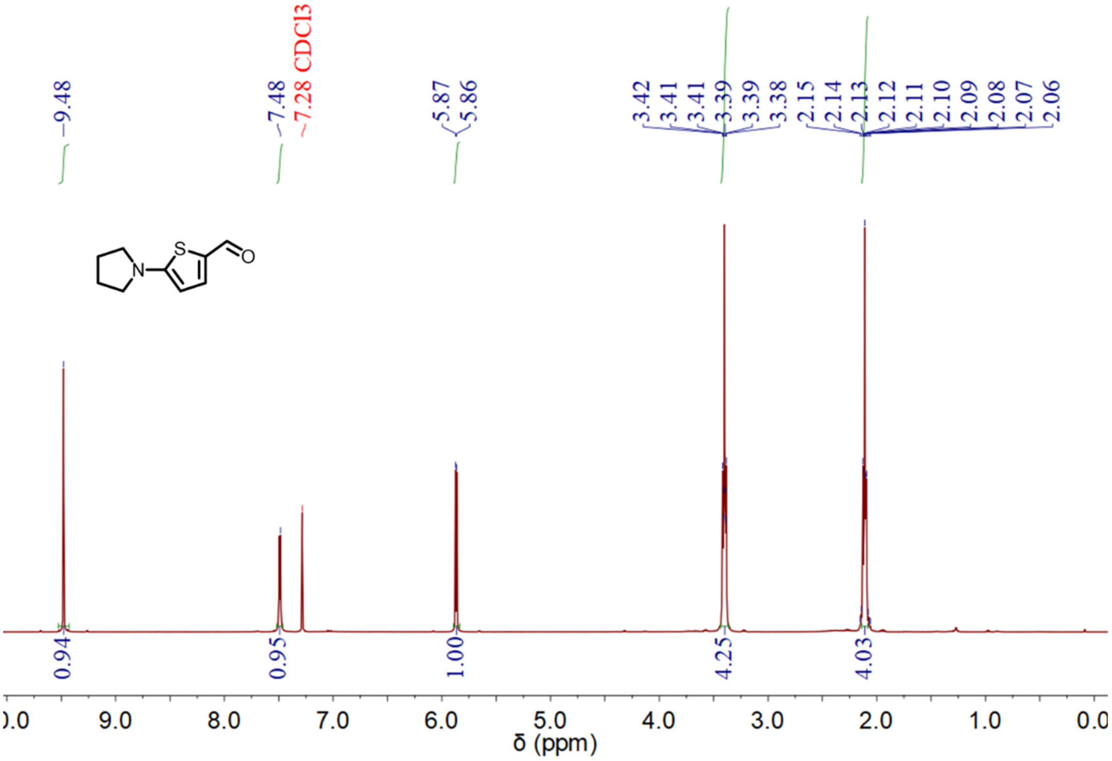

Figure S3. The ^{1}H NMR of compound 1 in $CDCl_3$.

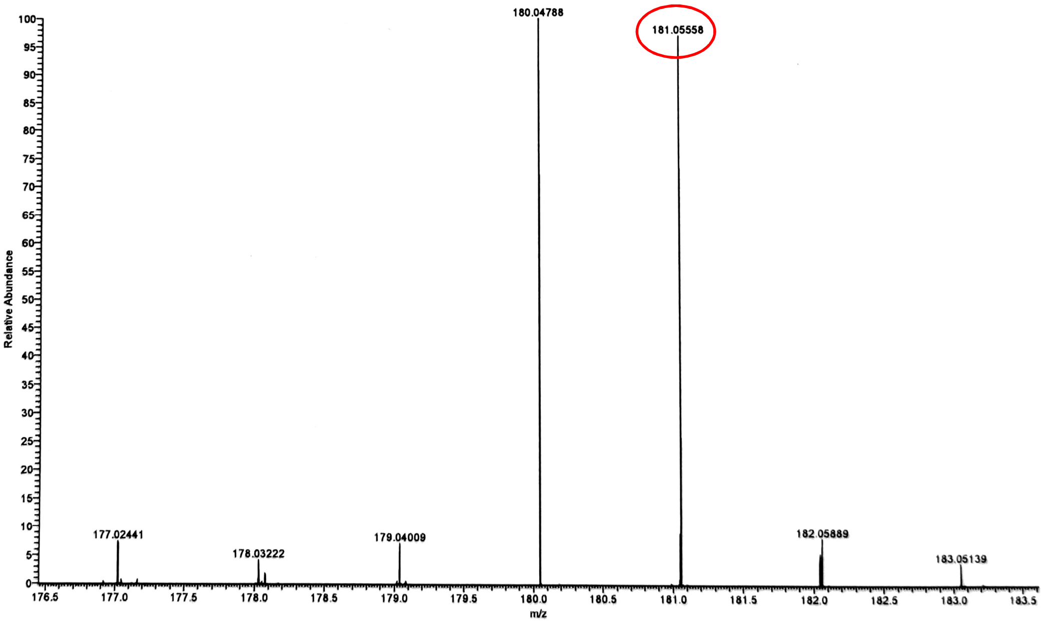

Figure S4. The mass spectrometry (EI-MS) of compound 1.

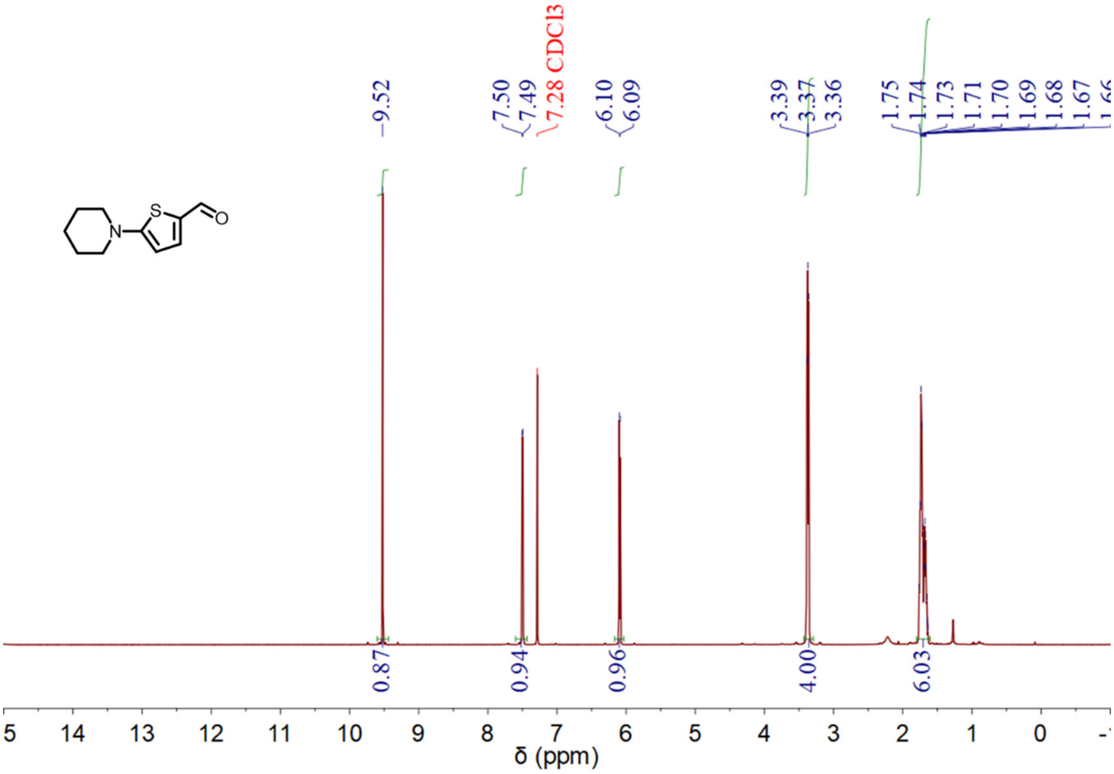

Figure S5. The ^{1}H NMR of compound 2 in $CDCl_3$.

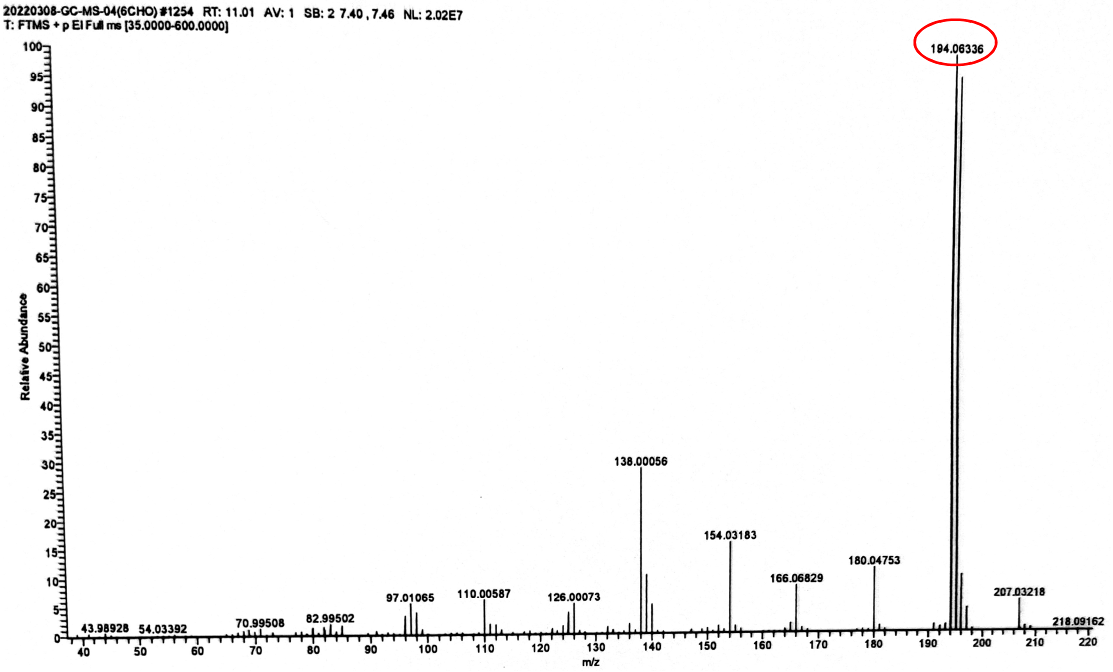

Figure S6. The mass spectrometry (EI-MS) of compound 2.

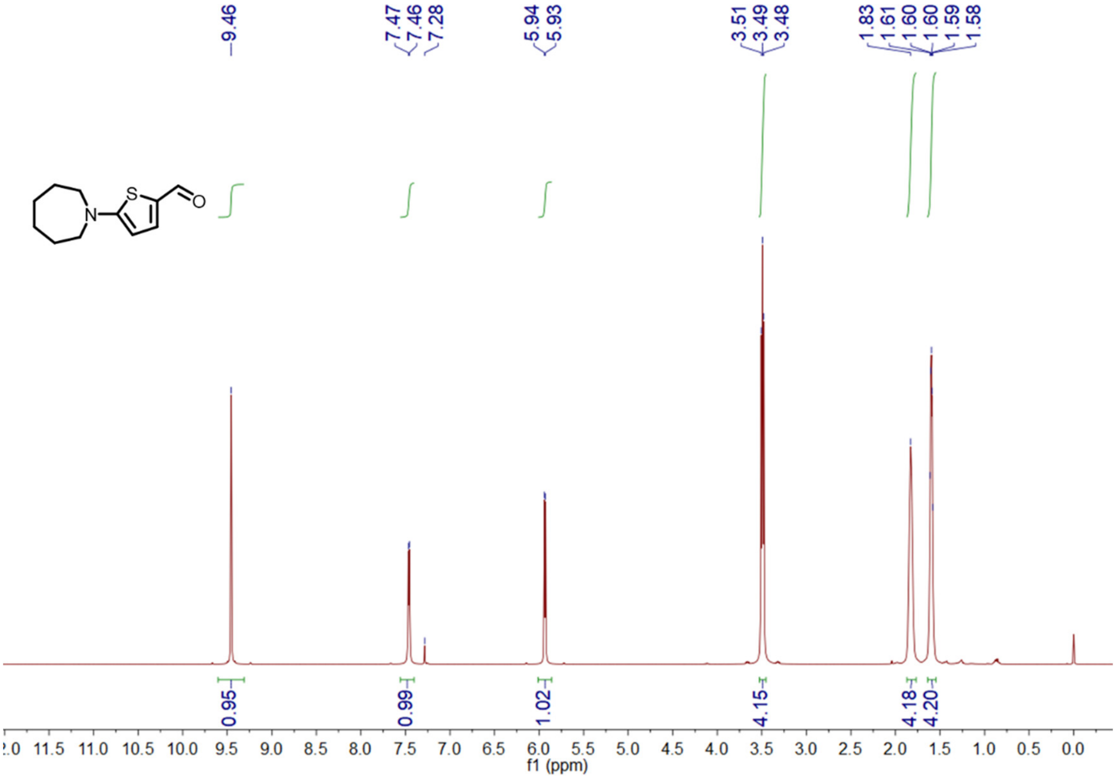

Figure S7. The ^{1}H NMR of compound 3 in $CDCl_3$.

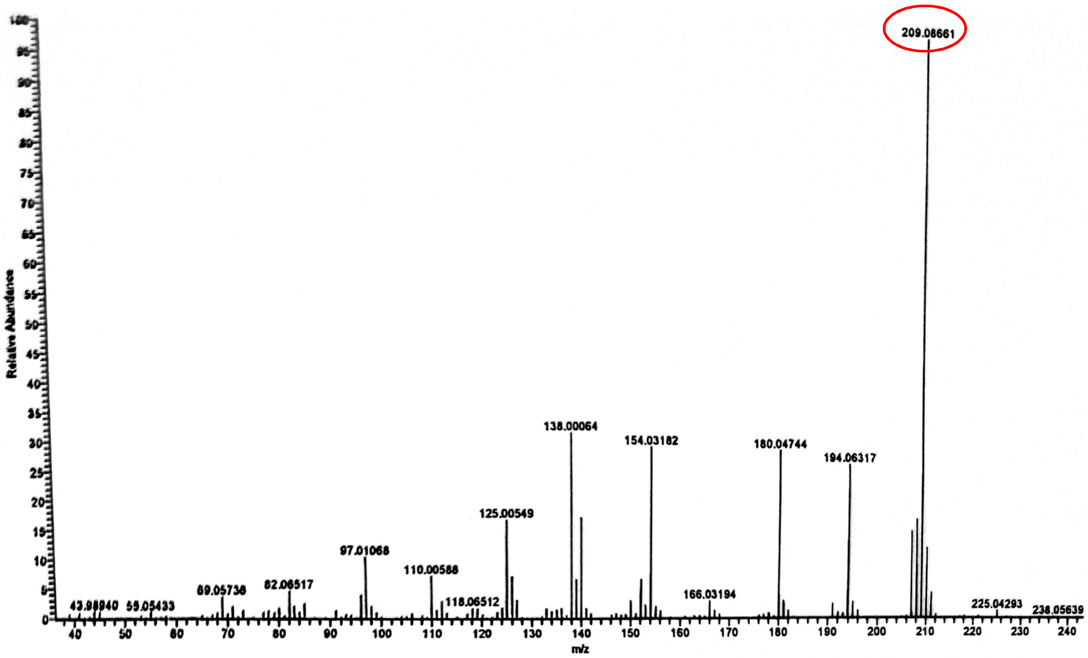

Figure S8. The mass spectrometry (EI-MS) of compound 3.

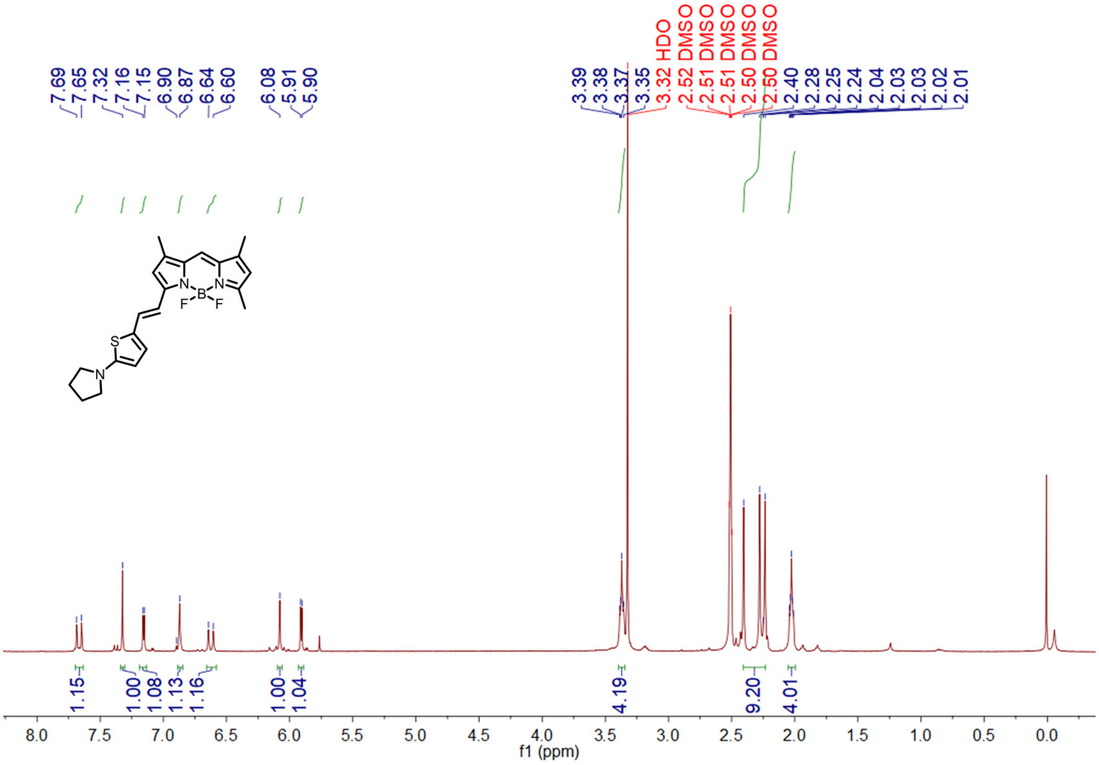

Figure S9. The ^{1}H NMR of TPyrBDP in DMSO-d_6.

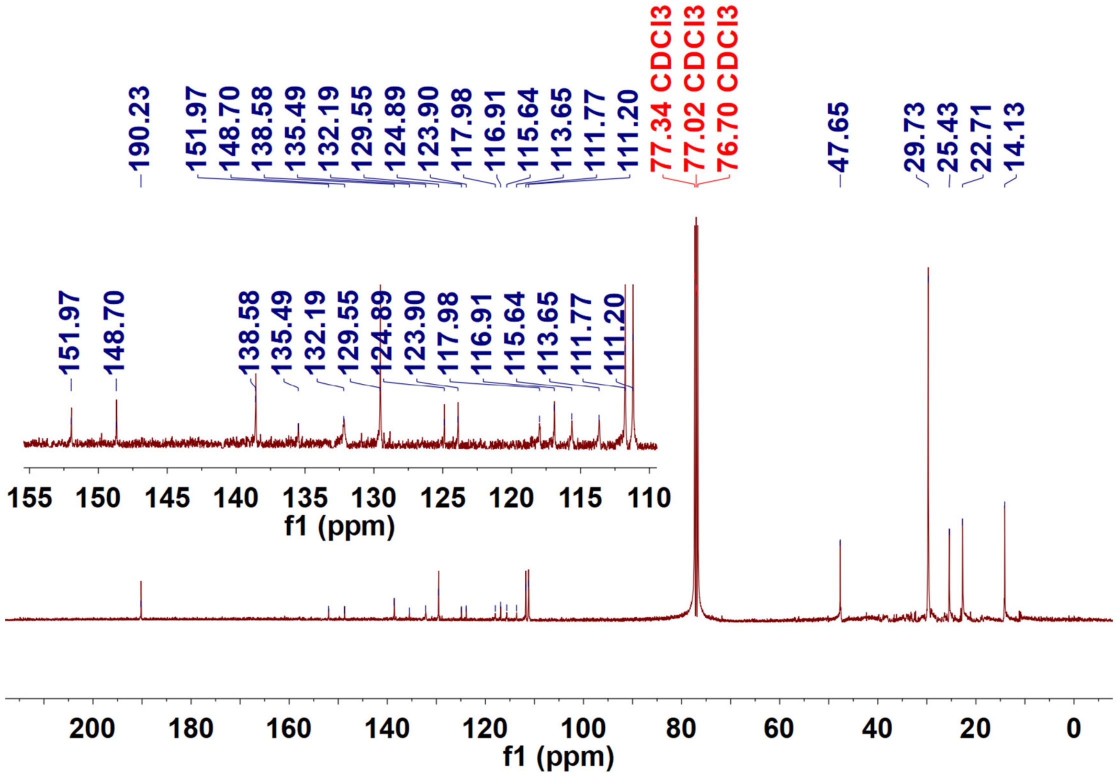

Figure S10. The ^{13}C NMR of TPyrBDP in $CDCl_3$.

Acquisition Parameter

Acquisition Mode	Single MS	Acquired Scans	2	Calibration Date	Mon Dec 20 05:26:50
Polarity	Positive	No. of Cell Fills	1	Data Acquisition Size	2097152
Broadband Low Mass	202.1 m/z	No. of Laser Shots	10	Data Processing Size	4194304
Broadband High Mass	800.0 m/z	Laser Power	18.0 lp	Apodization	Sine-Bell Multiplication
Source Accumulation	0.001 sec	Laser Shot Frequency	0.020 sec		
Ion Accumulation Time	0.100 sec				

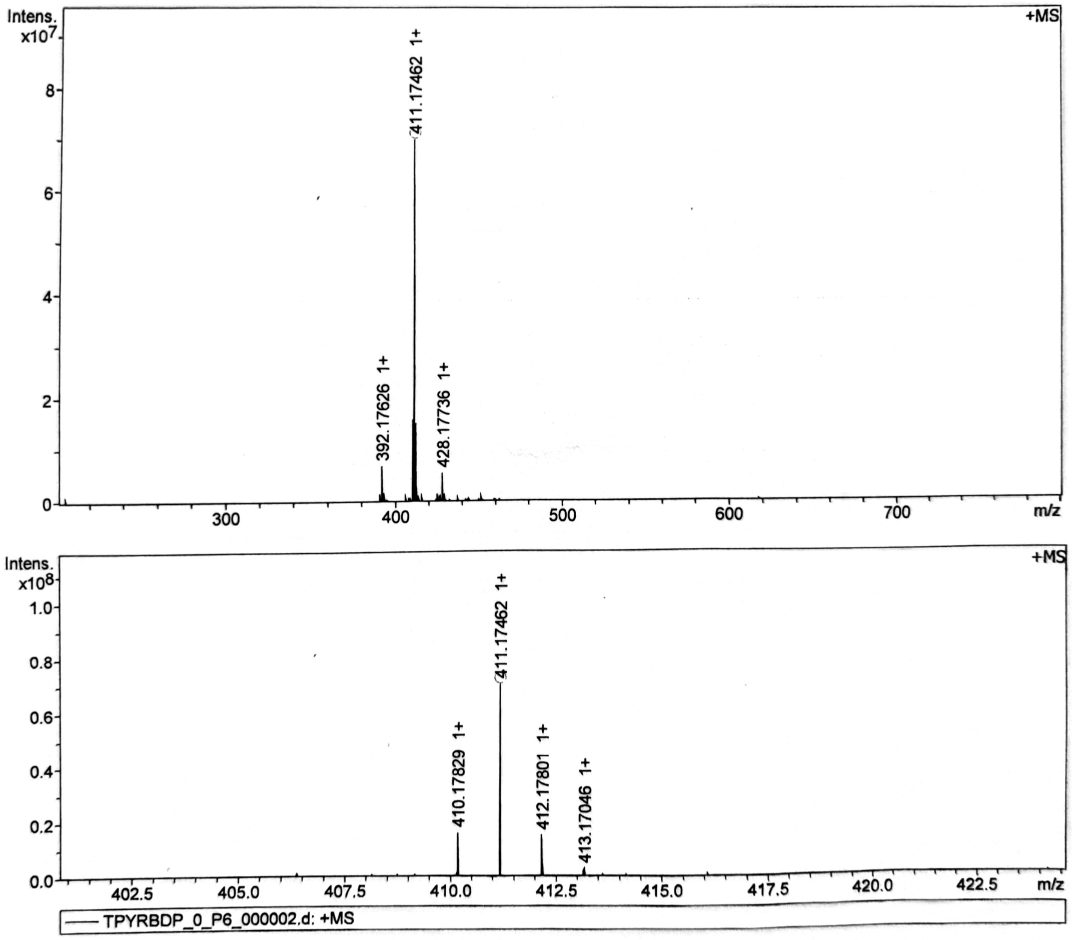

Meas. m/z	#	Ion Formula	Score	m/z	err [ppm]	Mean err [ppm]	mSigma	rdb	e⁻ Conf	N-Rule
411.174619	1	C22H24BF2N3S	100.00	411.175059	1.1	0.7	38.6	12.0	odd	ok

Figure S11. The HRMS (MALDI-TOF) of TPyrBDP.

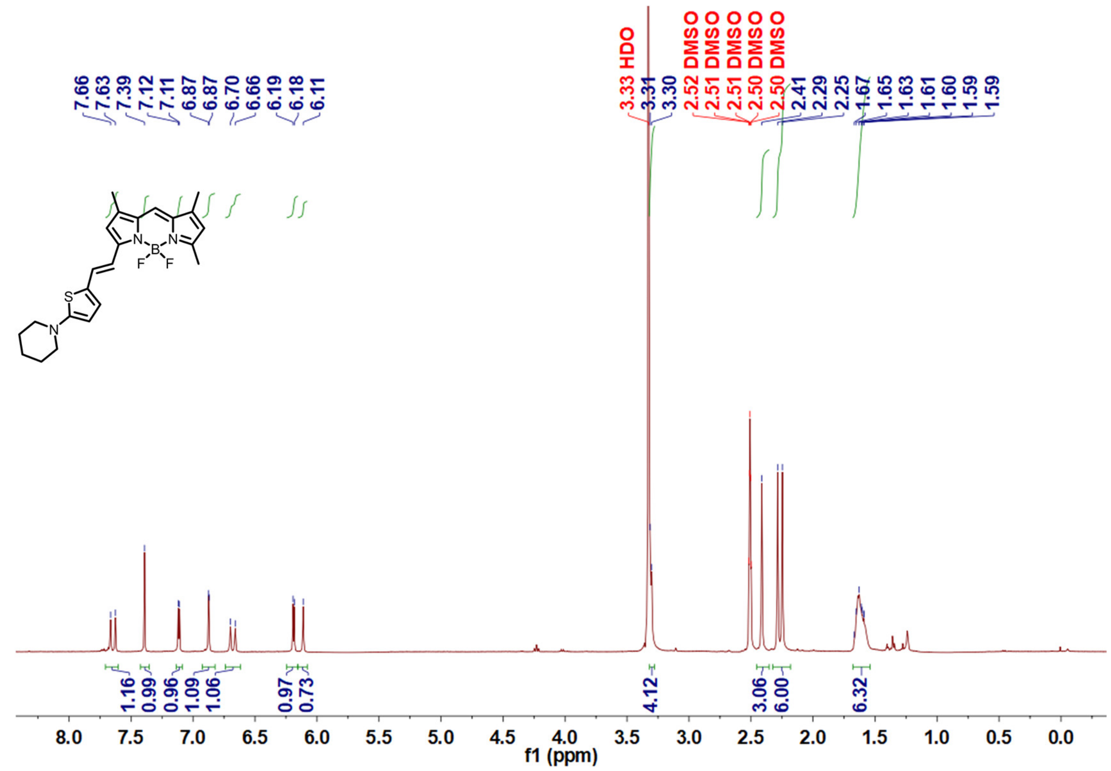

Figure S12. The ^{1}H NMR of TPipBDP in DMSO-d6.

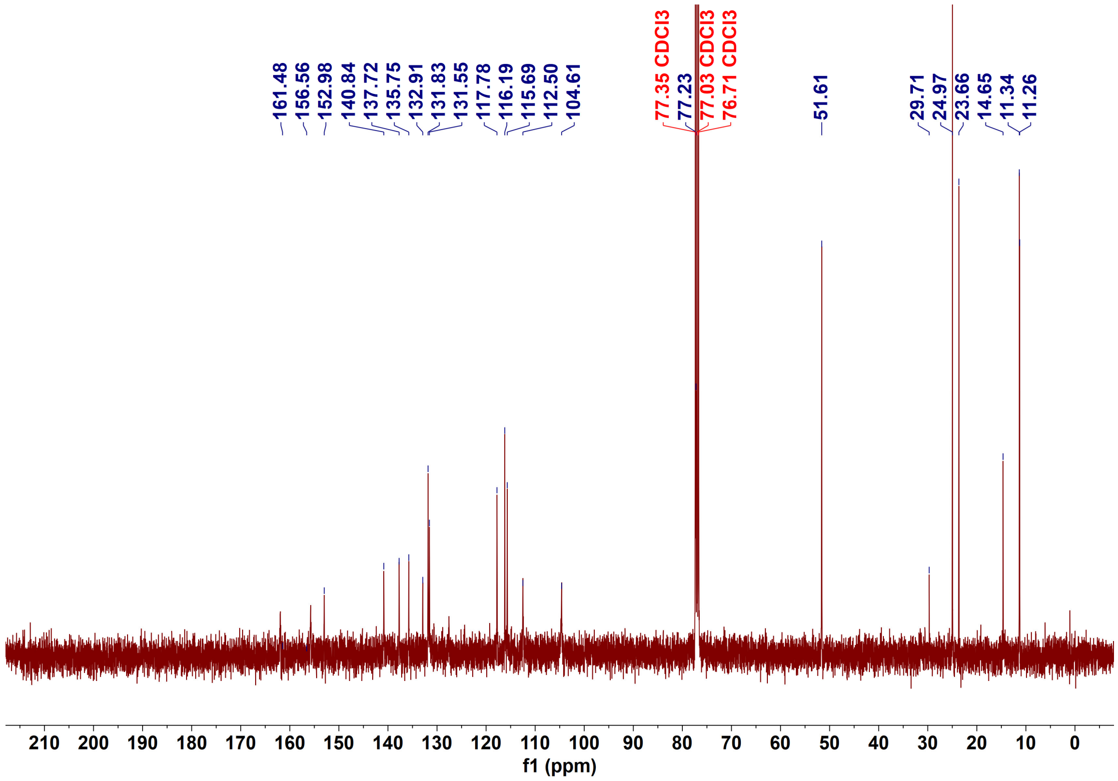

Figure S13. The ^{13}C NMR of TPipBDP in $CDCl_3$.

Acquisition Parameter

Acquisition Mode	Single MS	Acquired Scans	2	Calibration Date	Mon Dec 20 05:26:50
Polarity	Positive	No. of Cell Fills	1	Data Acquisition Size	2097152
Broadband Low Mass	202.1 m/z	No. of Laser Shots	10	Data Processing Size	4194304
Broadband High Mass	800.0 m/z	Laser Power	16.0 lp	Apodization	Sine-Bell Multiplication
Source Accumulation	0.001 sec	Laser Shot Frequency	0.020 sec		
Ion Accumulation Time	0.100 sec				

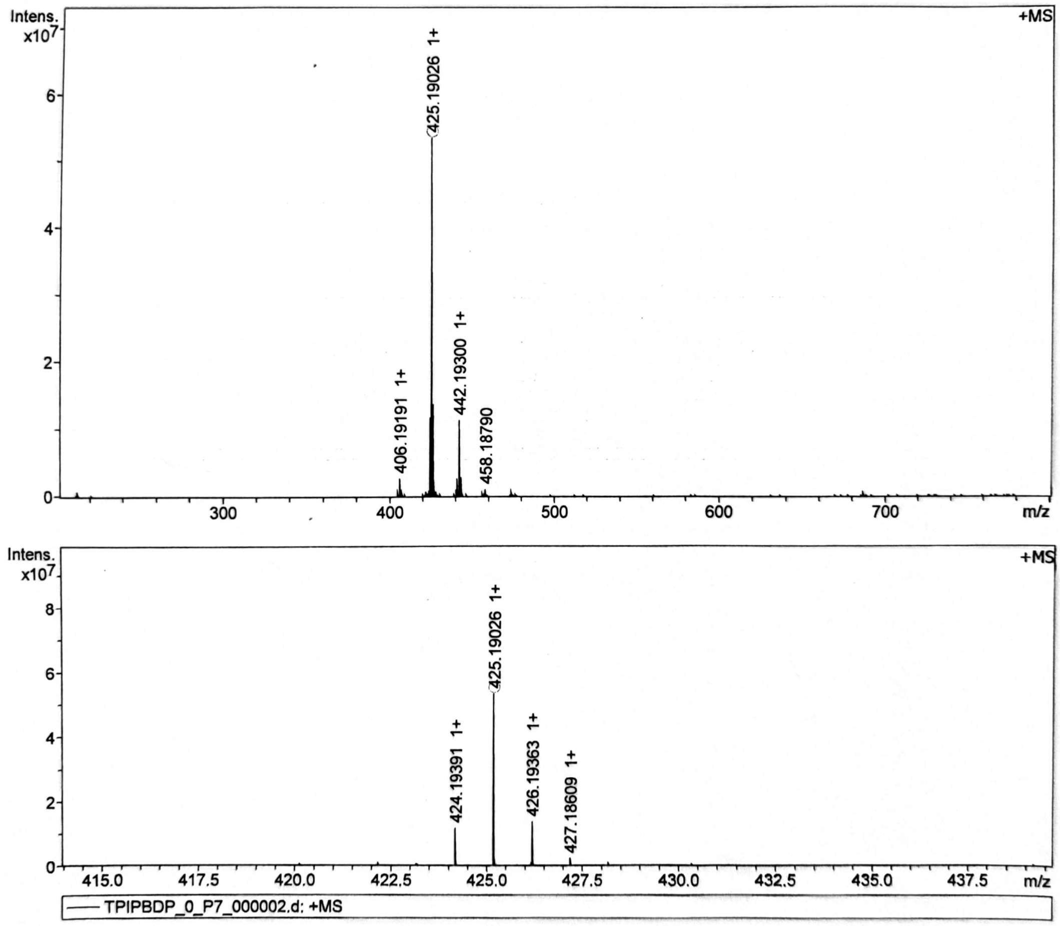

Meas. m/z	#	Ion Formula	Score	m/z	err [ppm]	Mean err [ppm]	mSigma	rdb	e⁻ Conf	N-Rule
425.190263	1	C23H26BF2N3S	100.00	425.190726	1.1	0.7	34.6	12.0	odd	ok

Figure S14. The HRMS (MALDI-TOF) of TPipBDP.

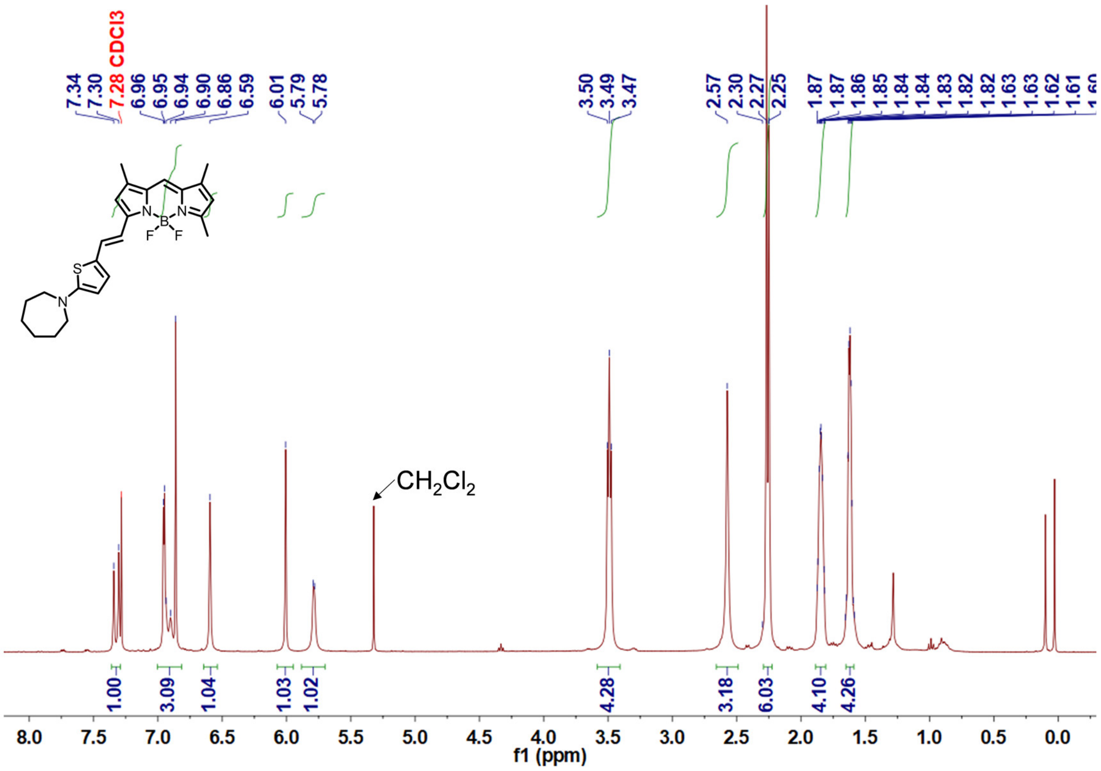

Figure S15. The ^{1}H NMR of THAIBDP in $CDCl_3$.

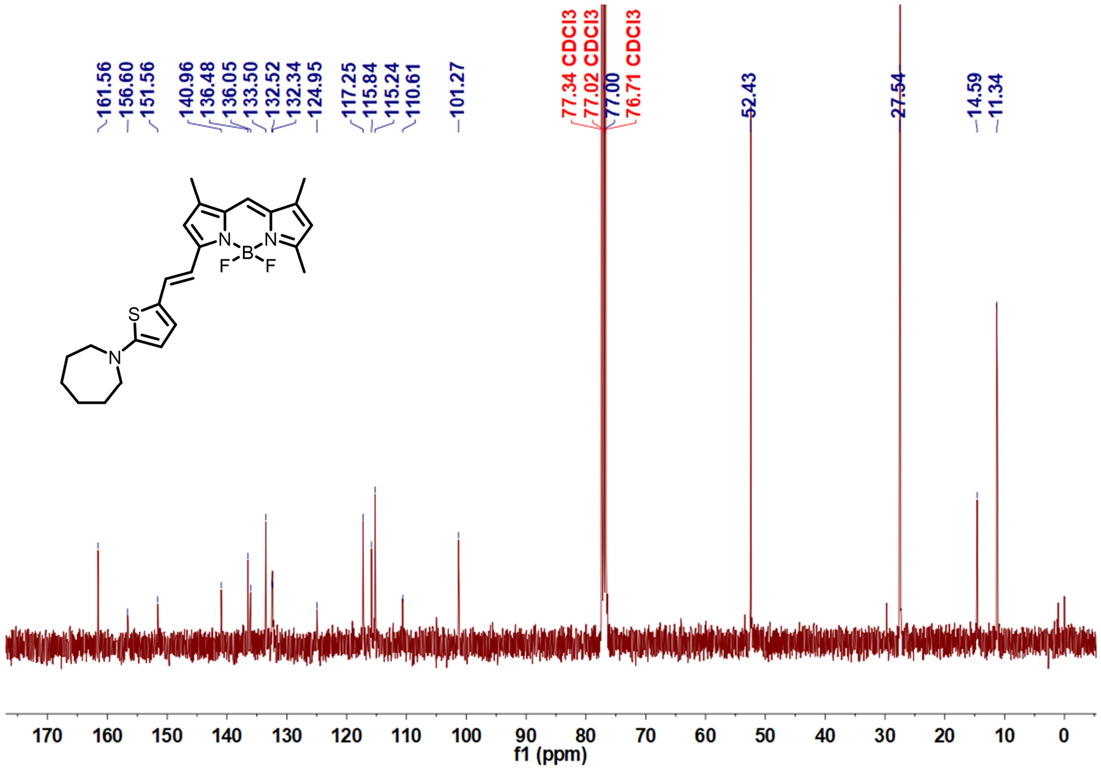

Figure S16. The ^{13}C NMR of THAIBDP.

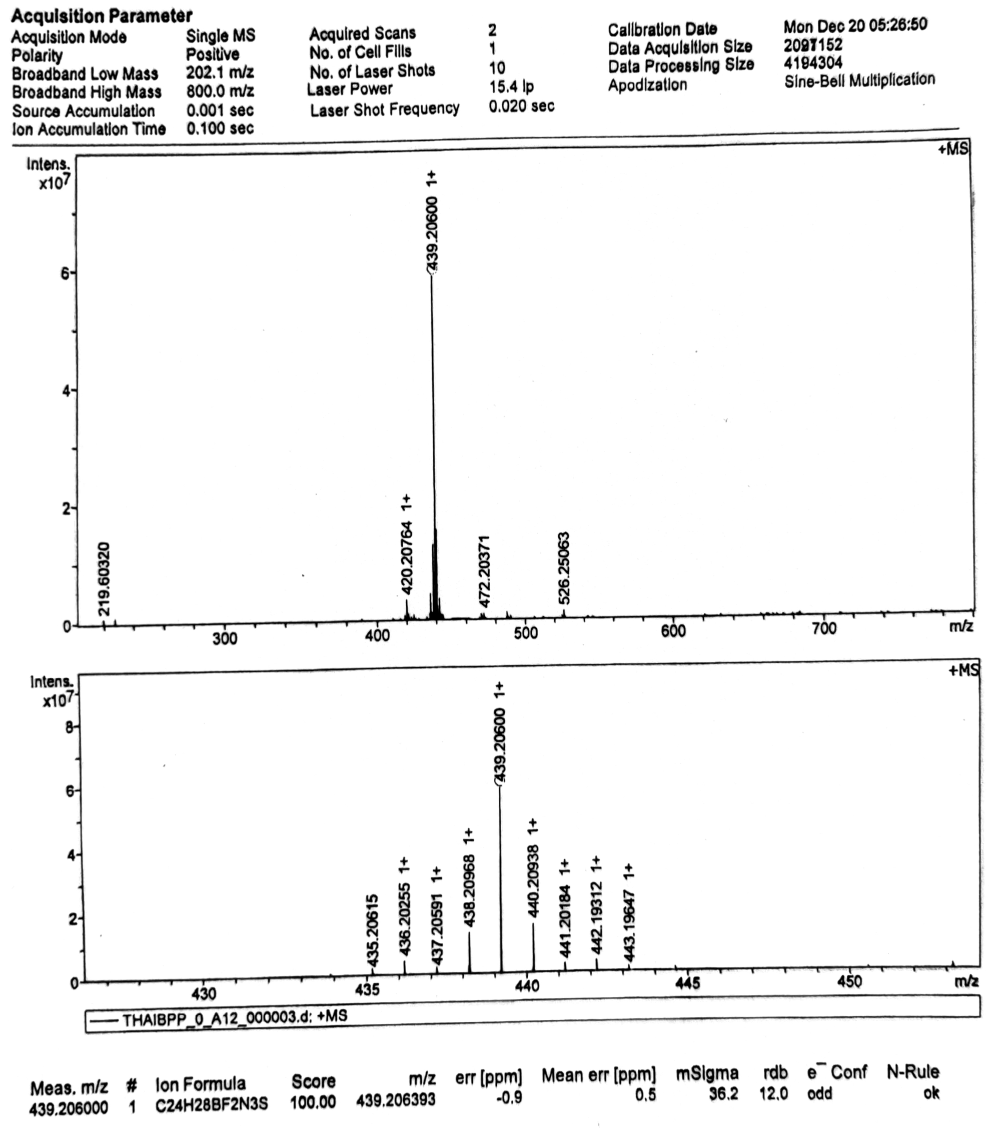

Figure S17. The HRMS (MALDI-TOF) of THAIBDP.

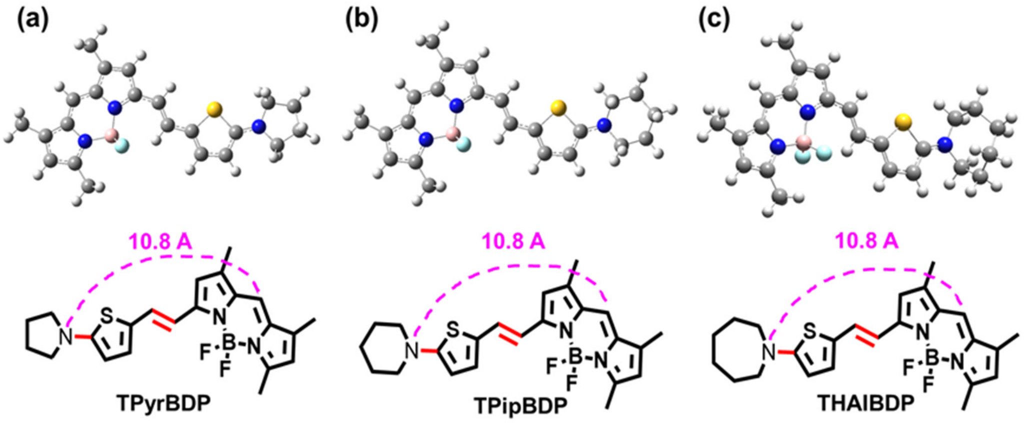

Figure S18. The molecular configuration optimized by Gaussian and distance from electron donor to acceptor. (a) TPyrBDP. (b) TPipBDP. (c) THAIBDP.

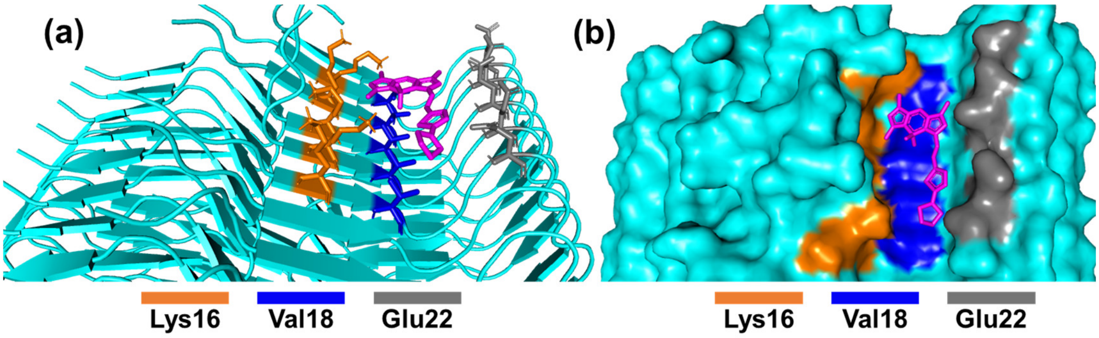

Figure S19. The overview of docking results of TPyrBDP in 5KK3 protein model. (a) The Licorice form of probe in Aβ_{1-42} aggregates model. (c) The molecular surface form corresponding to Figure a.

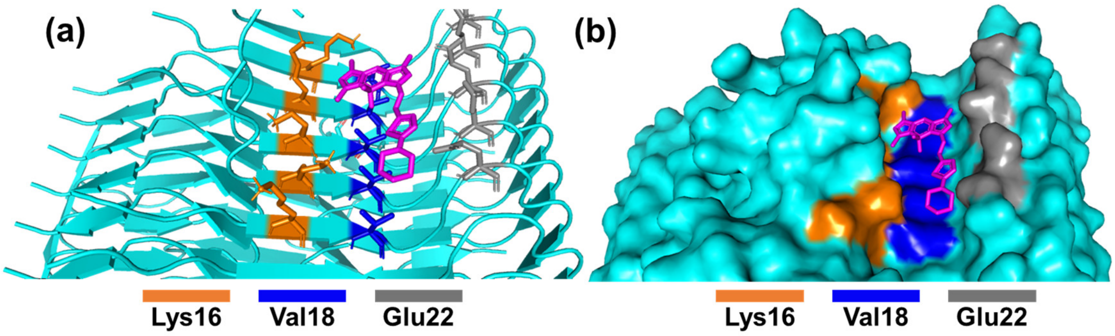

Figure S20. The overview of docking results of TPipBDP in 5KK3 protein model. (a) The Licorice form of probe in Aβ_{1-42} aggregates model. (c) The molecular surface form corresponding to Figure a.

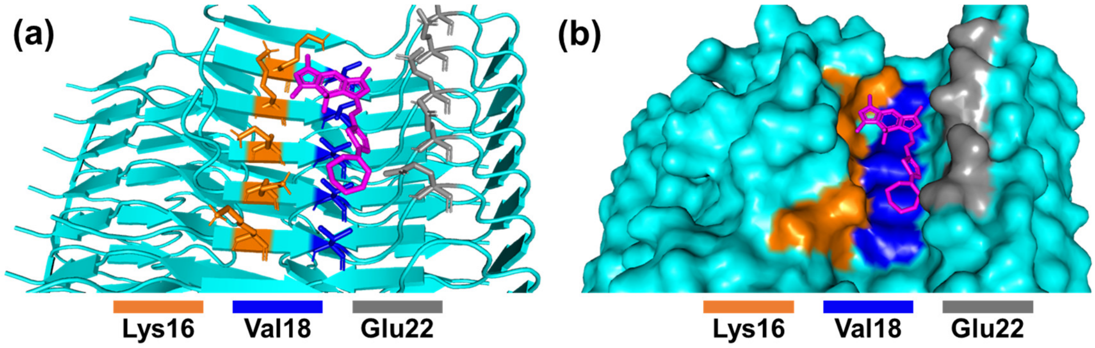

Figure S21. The overview of docking results of THAIBDP in 5KK3 protein model. (a) The Licorice form of probe in Aβ_{1-42} aggregates model. (c) The molecular surface form corresponding to Figure a.

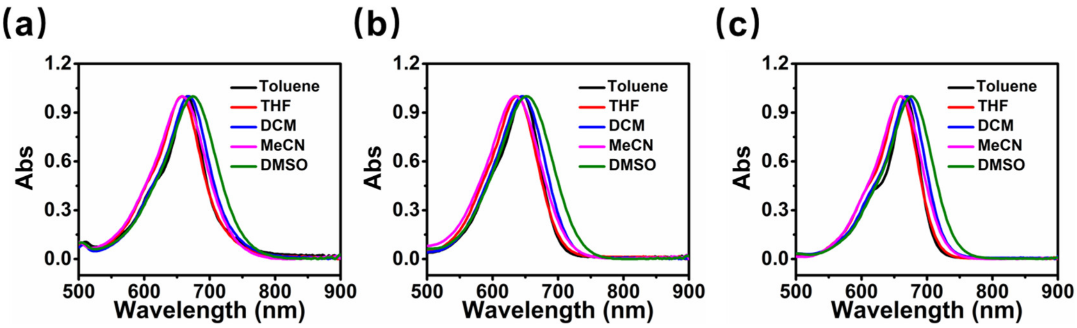

Figure S22. The absorption spectra of probes in different solvents. (a) TPyrBDP. (b) TPipBDP. (c) THAIBDP.

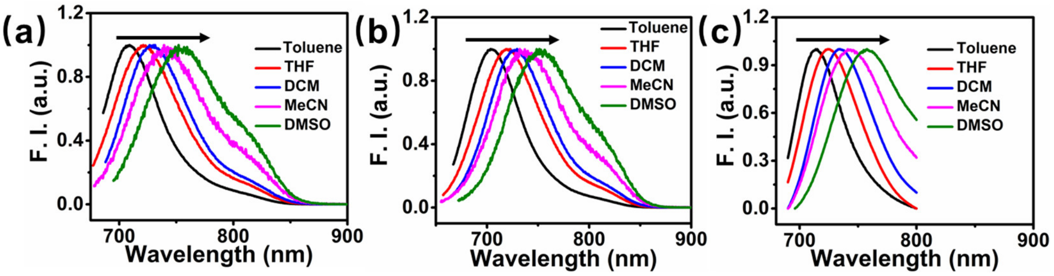

Figure S23. The emission spectra of probes in different solvents. (a) TPyrBDP. (b) TPipBDP. (c) THAIBDP.

Table S1. The spectral data of probes.

**Probes**	**Solvents**	**Ex [nm]**	**Em [nm][a]**	**Stokes shift [nm]**	**Em, with Aβ [nm][a]**	**Em shift, with Aβ [nm][a,d]**	**Fold[b]**	**ClogP[c]**	**Kd [nM]**
TPyrBDP	Toluene	667	709	42	693	+13	42.48	3.41	54.16±5.82
	THF	658	721	63					
	DCM	669	731	62					
	MeCN	659	739	80					
	DMSO	675	752	77					
TPipBDP	Toluene	647	704	57	692	-27	75.54	3.60	28.30±5.94
	THF	638	723	85					
	DCM	647	729	82					
	MeCN	636	732	96					
	DMSO	653	751	98					
THAIBDP	Toluene	668	714	46	701	-22	27.23	3.77	28.16±3.07
	THF	660	725	65					
	DCM	670	734	64					
	MeCN	660	741	81					
	DMSO	676	758	82					

(a) The emission wavelength (Em) was tested in 10% ethanol/PBS.

(b) The Fold was calculated according to the formula below.

Fold = FL intensity (probe+Aβ)/ FL intensity (probe)

(c) The clog *P* was calculated by online ALOGPS system with non-java interface (http://www.vcclab.org/lab/alogps/).

n.d. means not determined.

(d) "+" means a red shift wavelength, and "-" means a blue shift wavelength.

Table S2. Comparison of our probes with reported probes

	Probe	Structure	Kd	Reference
1	AOI-987	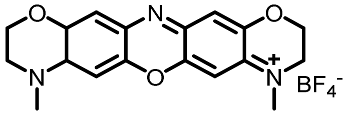	220 nM	(3)
2	NIAD-4	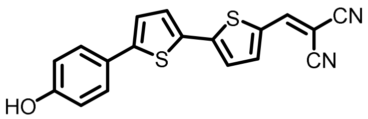	54.6 nM	(4)
3	QM-FN-SO_3	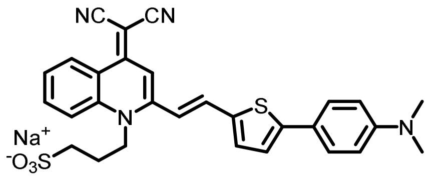	170 nM	(5)
4	DANIR 2c	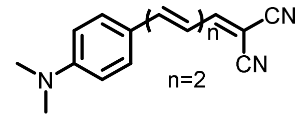	27 nM	(6)
5	CRANAD-2	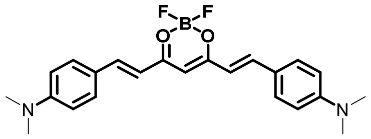	38.69 nM	(7)
6	BAP-1	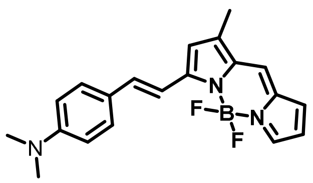	44.1 nM	(8)
7	BAP-2	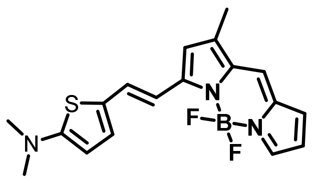	55 nM	(9)

8	BAP-3	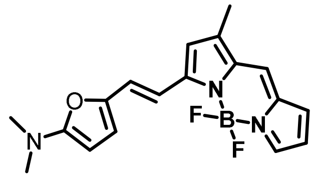	149 nM	(9)
9	BAP-4	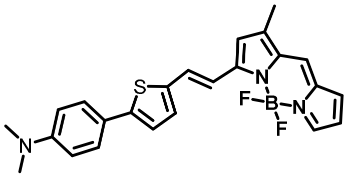	27 nM	(9)
10	BAP-5	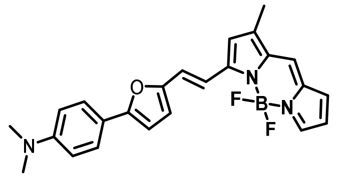	18 nM	(9)
11	EUA-1	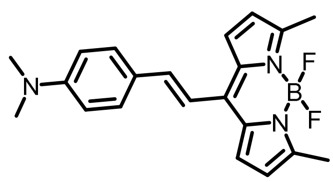	320 nM	(10)
12	EUA-2	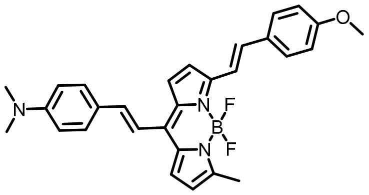	230 nM	(10)
13	EUA-4	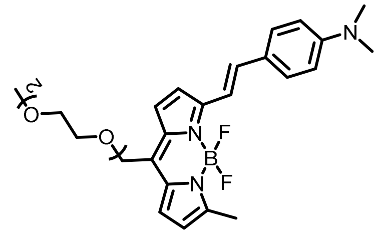	48.6 nM	(10)
14	ThT	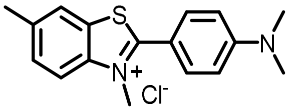	890 nM	(5)
15	QAD-1	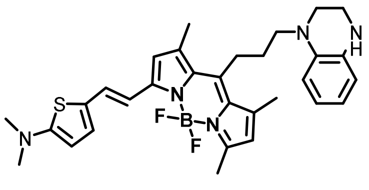	27 nM	(12)
16	ARCAM1	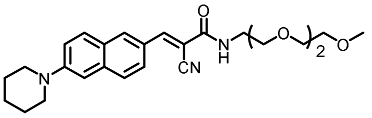	870 ± 280 nM	(12)

17	TPyrBDP	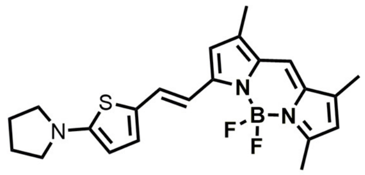	54.16 ± 5.82 nM	This work
18	TPipBDP	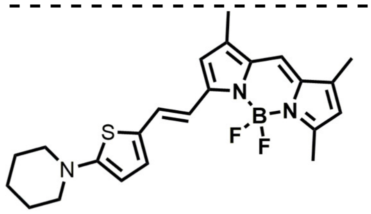	28.30 ± 5.94 nM	This work
19	THAIBDP	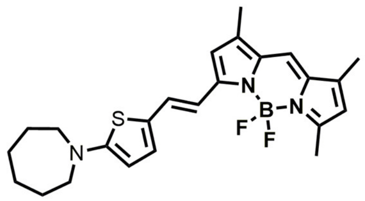	28.16 ± 3.07 nM	This work

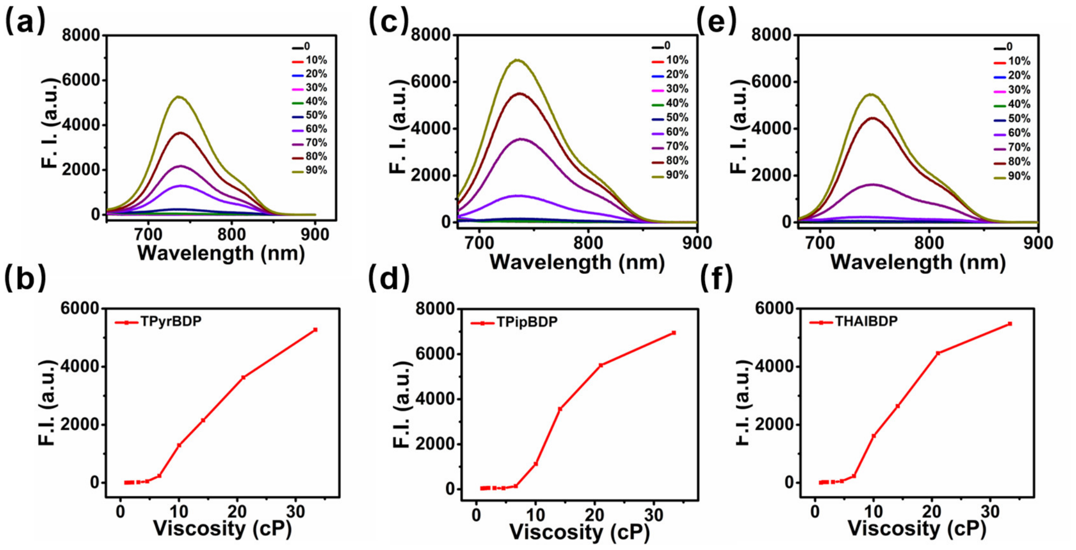

Figure S24. The viscosity response characteristics of BDPs. (a, b) TPyrBDP. (c, d) TPipBDP. (e, f) THAIBDP. The solvent was different ratios of 1,2-propanediol and water (v/v).

Table S3. The relationship between volume percentage and viscosity

Ratio (%)	0	10	20	30	40	50	60	70	80	90
Viscosity (cP)	1	1.42	2.02	3.06	4.57	6.65	10.04	14.15	21.04	33.38

NOTE: The data (T = 20 °C) is sourced from the 2005 ASHRAE Handbook: Fundamentals

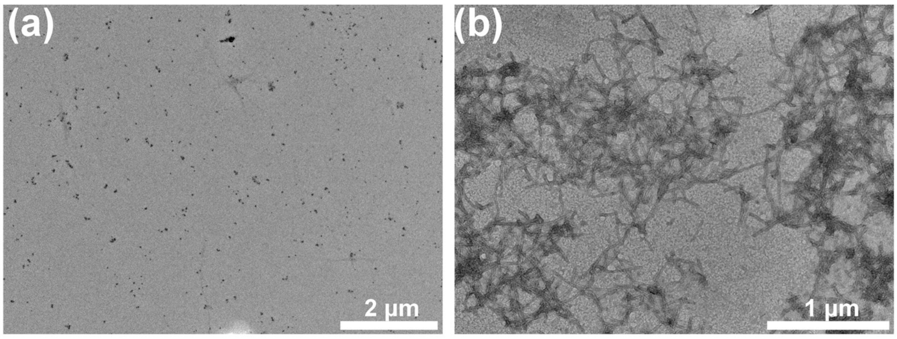

Figure S25. The TEM images of (a) Aβ_{42} oligomers and (b) Aβ_{42} aggregates.[13]

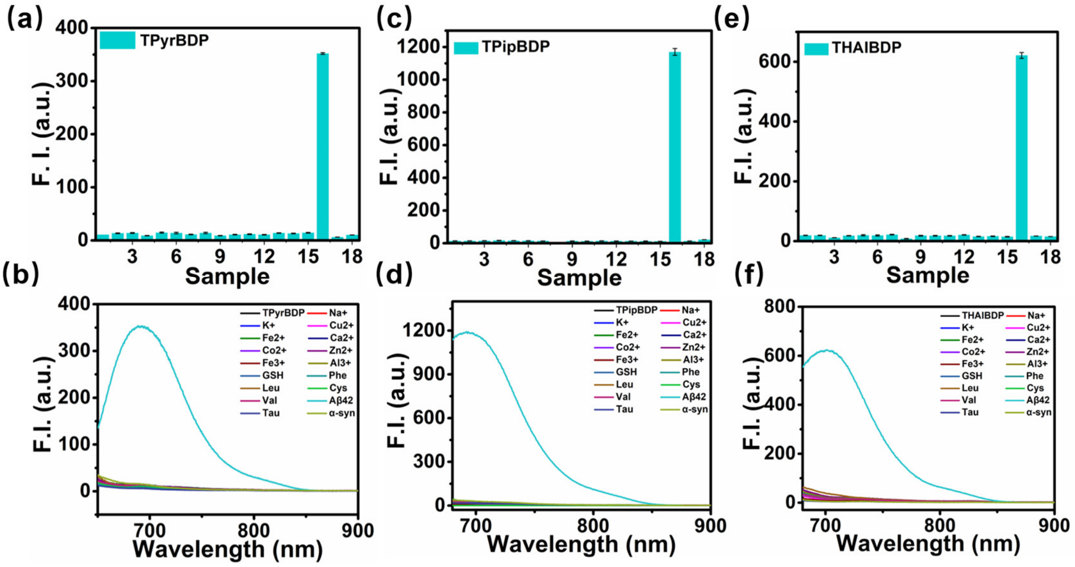

Figure S26. The selectivity of (a, b) TPyrBDP, (c, d) TPipBDP, (e, f) THAIBDP to different ions, amino acids, and Aβ_{42} aggregates. 1: probes; 2: Na^{+}; 3: K^{+}; 4: Cu^{2+}; 5: Fe^{2+}; 6: Ca^{2+}; 7: Co^{2+}; 8: Zn^{2+}; 9: Fe^{3+}; 10: Al^{3+}; 11: GSH; 12: Phe; 13: Leu; 14: Cys; 15: Val; 16: Aβ_{42} aggregates; 17: Tau; 18: α-synuclein.

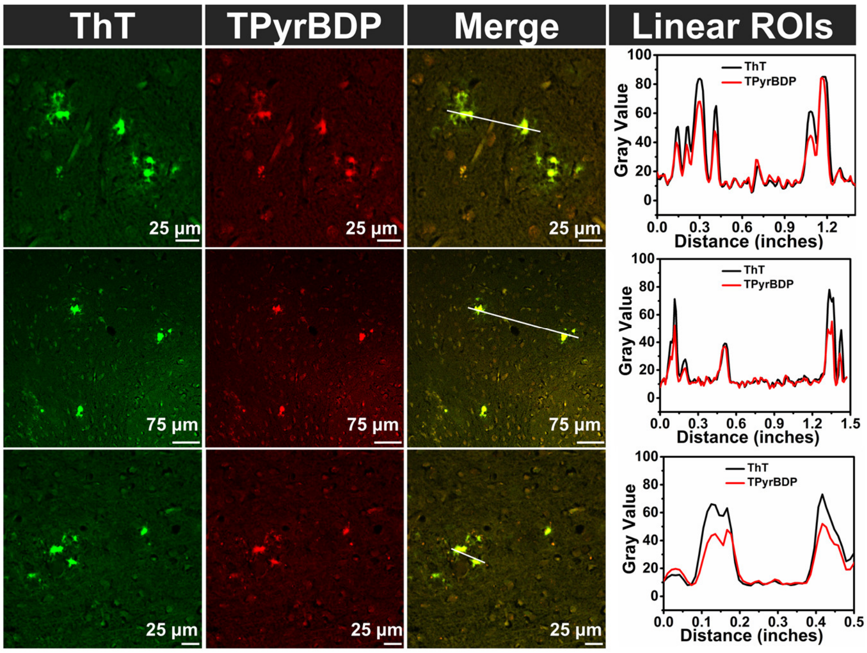

Figure S27. *In vitro* fluorescence staining results (TPyrBDP) for brain slices from Tg mice (C57BL/6J, APP/PS1, 13 months old, female). ThT: λ_{ex} = 488 nm, λ_{em} = 500-600 nm. TPyrBDP: λ_{ex} = 488 nm (60%) and 633 nm (30%), λ_{em} = 650-750 nm.

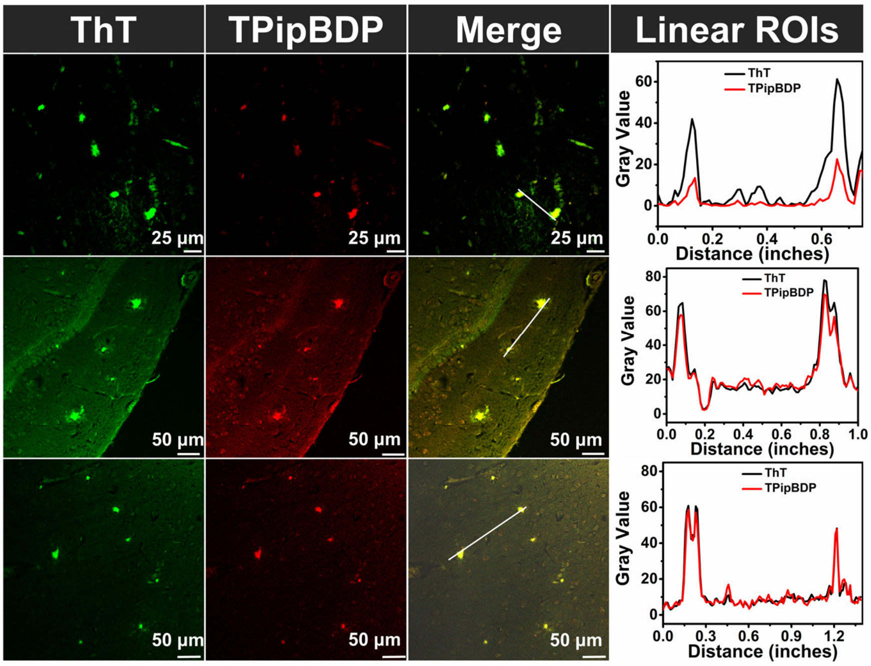

Figure S28. *In vitro* fluorescence staining results (TPipBDP) for brain slices from Tg mice (C57BL/6J, APP/PS1, 13 months old, female). ThT: λ_{ex} = 488 nm, λ_{em} = 500-600 nm. TPipBDP: λ_{ex} = 488 nm (60%) and 633 nm (30%), λ_{em} = 650-750 nm.

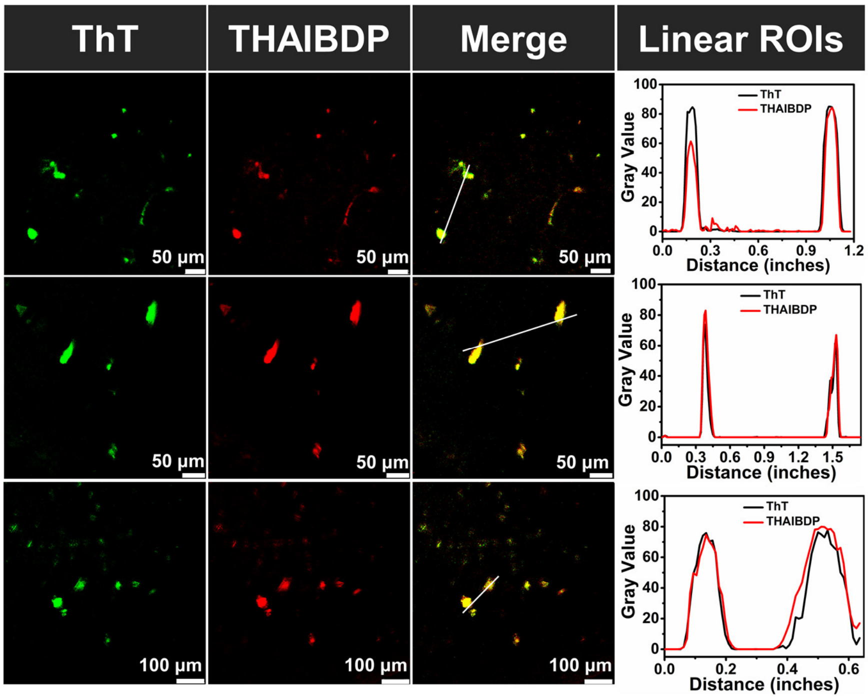

Figure S29. *In vitro* fluorescence staining results (THAIBDP) for brain slices from Tg mice (C57BL/6J, APP/PS1, 13 months old, female). ThT: λ_{ex} = 488 nm, λ_{em} = 500-600 nm. THAIBDP: λ_{ex} = 488 nm (60%) and 633 nm (30%), λ_{em} = 650-750 nm.

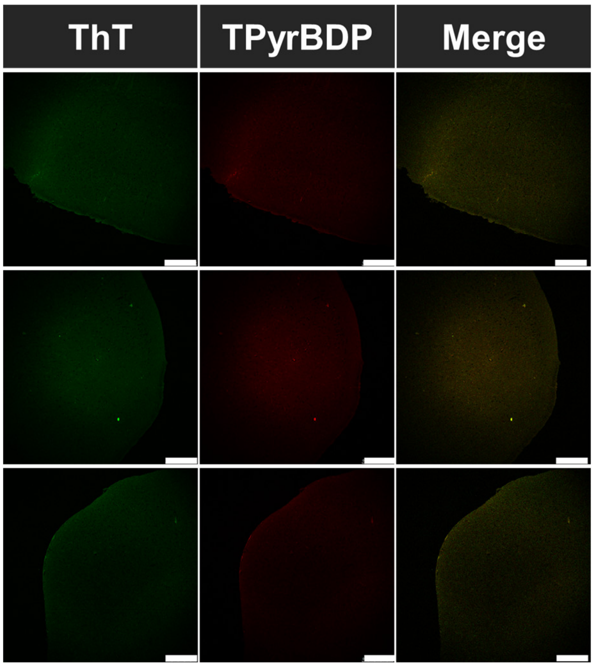

Figure S30. *In vitro* fluorescence staining results (TPyrBDP) for brain slices from WT mice (C57BL/6J, 13 months old, female). The scale bar is 250 μm. ThT: λ_{ex} = 488 nm, λ_{em} = 500-600 nm. TPyrBDP: λ_{ex} = 488 nm (60%) and 633 nm (30%), λ_{em} = 650-750 nm.

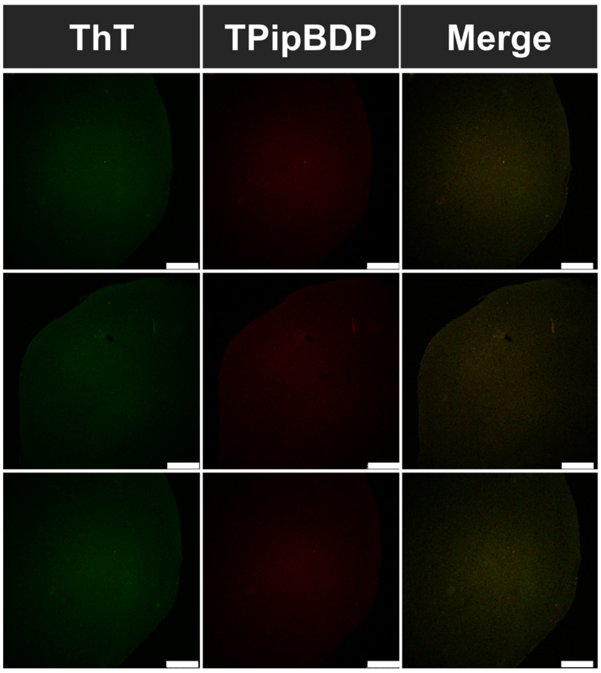

Figure S31. *In vitro* fluorescence staining results (TPipBDP) for brain slices from WT mice (C57BL/6J, 13 months old, female). The scale bar is 250 µm. ThT: λ_{ex} = 488 nm, λ_{em} = 500-600 nm. TPipBDP: λ_{ex} = 488 nm (60%) and 633 nm (30%), λ_{em} = 650-750 nm.

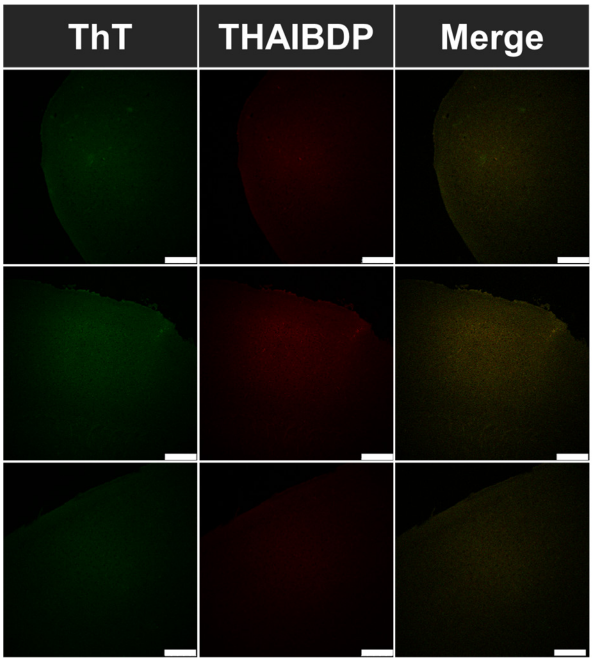

Figure S32. *In vitro* fluorescence staining results (TPipBDP) for brain slices from WT mice (C57BL/6J, 13 months old, female). The scale bar is 250 µm. ThT: λ_{ex} = 488 nm, λ_{em} = 500-600 nm. THAIBDP: λ_{ex} = 488 nm (60%) and 633 nm (30%), λ_{em} = 650-750 nm.

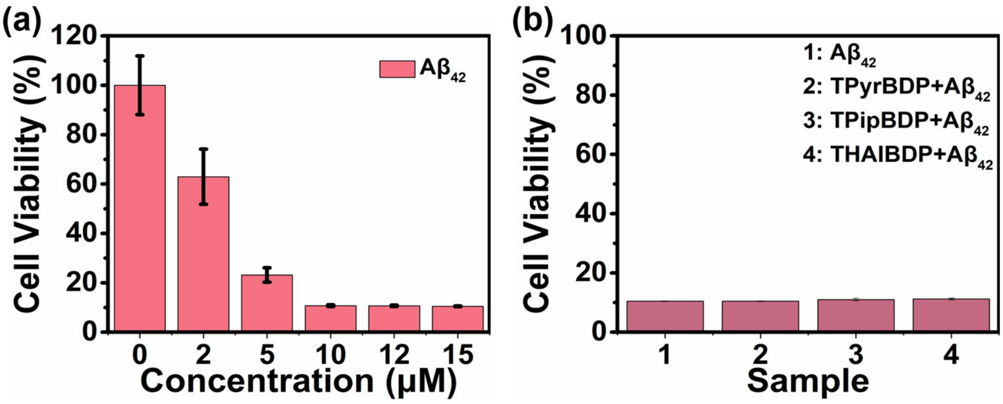

Figure S33. (a) Cytotoxicity of $A\beta_{42}$. (b) Cytotoxicity of "Probe + $A\beta_{42}$".

Discussion of cytotoxicity

We also evaluated the cytotoxicity of $A\beta_{1\text{-}42}$ aggregates with different concentrations. As shown in Figure S33a, the cytotoxicity was below 30% in 24 h when the concentration of $A\beta_{1\text{-}42}$ aggregates was 5 μM. PC12 cell viability was below 20% in 24 h when the concentration of $A\beta_{1\text{-}42}$ aggregates was higher than 10 μM. The results illustrated that $A\beta_{1\text{-}42}$ aggregates have great cytotoxicity when the concentration was higher than 5 μM. We further evaluated the cytotoxicity of "probe + $A\beta_{1\text{-}42}$ aggregates". The concentration of $A\beta_{1\text{-}42}$ aggregates was fixed as 10 μM. The concentration of probes was fixed as 5 μM. The probes had low cytotoxicity when their concentrations were 5 μM (Figure 5a). As shown in Figure S33b, regardless of the probe molecules, the cell viability was less than 20%. The above results indicated that $A\beta_{1\text{-}42}$ aggregates had obvious cytotoxicity, and our probes showed low cytotoxicity for PC12 cells

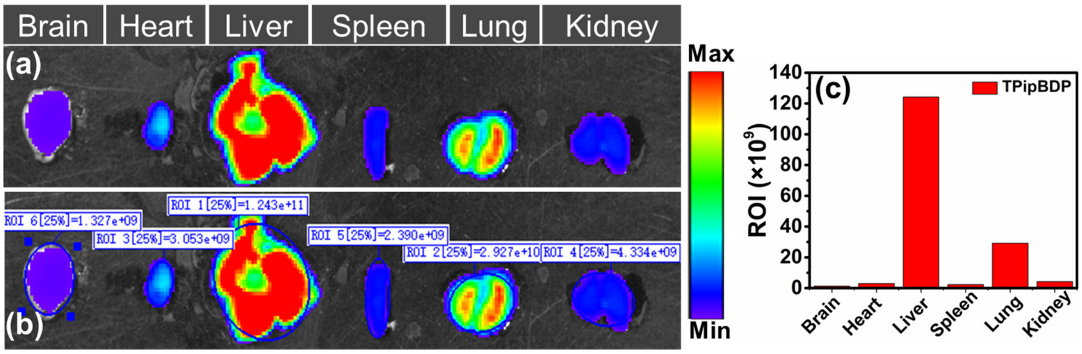

Figure S34. (a) The biodistribution of TPipBDP (1 mg/kg, 40% 1,2-propanediol/40% PBS/20% DMSO) after intravenous injection. The data was processed without ROI. (b) The biodistribution of TPipBDP (1 mg/kg, 40% 1,2-propanediol/40% PBS/20% DMSO)

after intravenous injection. The data was processed with ROI. (c) The semi-quantitative data of biodistribution. The organs were separated after 5 min and imaging immediately.

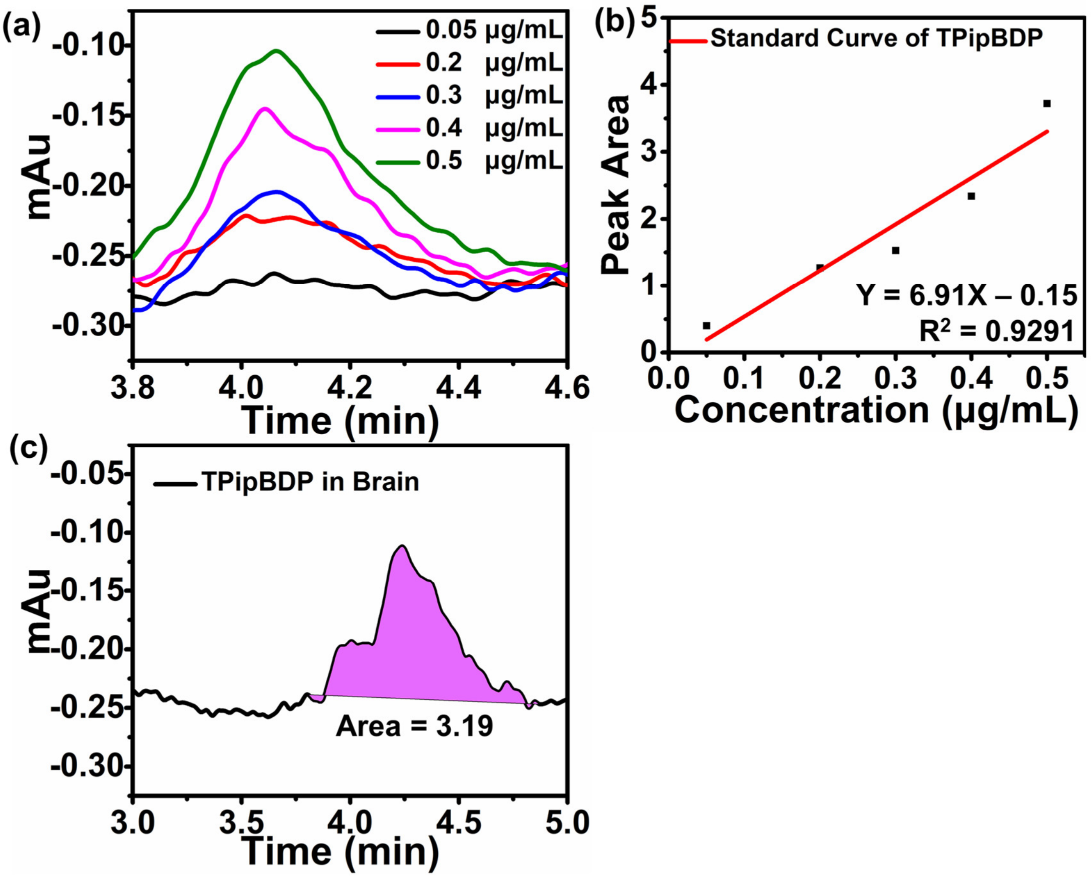

Figure S35. The BBB penetration rate of TPipBDP. (a) The HPLC chromatogram curve of TPipBDP. (b) The standard curve of TPipBDP. (c) The representative peak area of TPipBDP in the brain.

Table S4. Uptake of TPipBDP in the brains of KM mice

Probe	Concentration [μg / mL]	Intravenous Injection Volume [μL]	HPLC injection volume [μL]	Peak Area	The quality of the probe in the brain [μg]	Wet weight of brain [g]	Uptake [% ID/g]	Mean Uptake [% ID/g]
TPipBDP	150	100	20	1.48	0.71	0.42	6.67	10.38 ± 4.11
TPipBDP	150	100	20	3.19	1.45	0.42	9.67	
TPipBDP	150	100	20	4.96	2.22	0.42	14.80	

References

(1) Liangxing Wu, Kevin Burgess. A new synthesis of symmetric boraindacene (BODIPY) dyes. *Chem. Commun.*, **2008**, 4933-4935.

(2) Joydev Hatai, Leila Motiei, David Margulies. Analyzing Amyloid Beta Aggregates with a Combinatorial Fluorescent Molecular Sensor. *J. Am. Chem. Soc.* **2017**, 139, 6, 2136–2139.

(3) Hintersteiner, M.; Enz, A.; Frey, P.; Jaton, A.-L.; Kinzy, W.; Kneuer, R.; Neumann, U.; Rudin, M.; Staufenbiel, M.; Stoeckli, M.In Vivo Detection of Amyloid-β Deposits by Near-Infrared Imaging Using an Oxazine-Derivative Probe. *Nat. Biotechnol.* **2005**, *23*, 577–583

(4) Nesterov, E. E.; Skoch, J.; Hyman, B. T.; Klunk, W. E.; Bacskai, B. J.; Swager, T. M. In Vivo Optical Imaging of Amyloid Aggregates in Brain: Design of Fluorescent Markers. Angew. Chem., Int. Ed. Engl. **2005**, 44 (34), 5452−5456.

(5) Wei Fu, Chenxu Yan, Zhiqian Guo, Jingjing Zhang, Haiyan Zhang*, He Tian, and Wei-Hong Zhu* Rational Design of Near-Infrared Aggregation-Induced-Emission-Active Probes: In Situ Mapping of Amyloid-β Plaques with Ultrasensitivity and High-Fidelity. *J. Am. Chem. Soc.* **2019**, 141, 7, 3171-3177

(6) Cui, M.; Ono, M.; Watanabe, H.; Kimura, H.; Liu, B.; Saji, H. Smart Near-Infrared Fluorescence Probes with Donor–Acceptor Structure for In Vivo Detection of β-Amyloid Deposits. *J. Am. Chem. Soc.* **2014**, *136*, 3388– 3394

(7) Ran, C.; Xu, X.; Raymond, S. B.; Ferrara, B. J.; Neal, K.; Bacskai, B. J.; Medarova, Z.; Moore, A. Design, Synthesis, and Testing of Difluoroboron-Derivatized Curcumins as Near-Infrared Probes for In Vivo Detection of Amyloid-β Deposits. *J. Am. Chem. Soc.* **2009**, *131*, 15257– 15261

(8) Ono, M.; Watanabe, H.; Kimura, H.; Saji, H. BODIPY-Based Molecular Probe for Imaging of Cerebral β-Amyloid Plaques. *ACS Chem. Neurosci.* **2012**, *3*, 319– 324

(9) Watanabe, H.; Ono, M.; Matsumura, K.; Yoshimura, M.; Kimura, H.; Saji, H. Molecular Imaging of β-Amyloid Plaques with Near-Infrared Boron Dipyrromethane (BODIPY)-Based Fluorescent Probes. *Mol. Imaging* **2013**, *12*, 338– 347.

(10) Sozmen, F.; Kolemen, S.; Kumada, H.-O.; Ono, M.; Saji, H.; Akkaya, E. U. Designing BODIPY-Based Probes for Fluorescence Imaging of β-Amyloid Plaques. *RSC Adv*. **2014**, 4 (92), 51032− 51037.

(11) Wenming Ren, Jingjing Zhang, Cheng Peng, Huaijiang Xiang, Jingjing Chen, Chengyuan Peng, Weiliang Zhu, Ruimin Huang, Haiyan Zhang, and Youhong Hu. Fluorescent Imaging of β-Amyloid Using BODIPY Based Near-Infrared Off–On Fluorescent Probe. *Bioconjugate Chemistry* **2018**, 29, 10, 3459-3466

(12) Guan, Y.; Cao, K. J.; Cantlon, A.; Elbel, K.; Theodorakis, E. A.; Walsh, D. M.; Yang, J.; Shah, J. V. *ACS Chem. Neurosci.* **2015**, 6, 9, 1503–1508.

(13) J. Hatai, L. Motiei, and D. Margulies. Analyzing Amyloid Beta Aggregates with a Combinatorial Fluorescent Molecular Sensor. *J. Am. Chem. Soc.* **2017**, 139, 6, 2136–2139.
